# Chronic attenuation of brain leptin signalling is associated with early metabolic dysfunction in lean rats

**DOI:** 10.1113/JP290832

**Published:** 2026-05-07

**Authors:** Cristina Pintado, Lorena Mazuecos, Óscar Gómez‐Torres, Beatriz Merino, Elena Casanueva‐Álvarez, Blanca Rubio, Emma Burgos‐Ramos, Sara Artigas‐Jerónimo, Eduardo Moltó, Inés María Ramos, Justa María Poveda, Germán Perdomo, Irene Cózar‐Castellano, Carmen Arribas, Ernesto Bernal‐Mizrachi, Antonio Andrés, Nilda Gallardo

**Affiliations:** ^1^ Biochemistry Section, Faculty of Environmental Sciences and Biochemistry University of Castilla‐La Mancha (UCLM) Toledo Spain; ^2^ DOE Research Group, Institute of Biomedicine IDISCAM Spain; ^3^ Biochemistry Section, Faculty of Chemical Sciences and Technologies University of Castilla‐La Mancha (UCLM) Ciudad Real Spain; ^4^ Unit of Excellence, Institute of Molecular Biology and Genetics (IBGM) University of Valladolid‐CSIC Valladolid Spain; ^5^ Department of Analytical Chemistry and Food Technology Regional Institute for Applied Scientific Research (IRICA), Faculty of Chemical Sciences and Technologies University of Castilla‐La Mancha (UCLM) Ciudad Real Spain; ^6^ Geriatric Research Education and Clinical Center Veterans Affairs Medical Center Miami FL USA; ^7^ Division of Endocrinology, Diabetes, and Metabolism, Department of Medicine University of Miami Miller School of Medicine Miami FL USA

**Keywords:** amino acid metabolism, brain leptin signalling, glucagon resistance, liver–α‐cell axis, neuroendocrine regulation, prediabetes

## Abstract

**Abstract:**

Brain leptin signalling plays a central role in the regulation of energy balance and glucose homeostasis, yet its contribution to early metabolic dysfunction preceding overt obesity remains uncertain. In the present study, we examined the metabolic consequences of sustained attenuation of central leptin receptor signalling in lean rats. Adult animals received chronic i.c.v. infusion of a rat‐specific leptin receptor antagonist (SLA) or vehicle for 21 days. SLA administration increased food intake with modest gains in body weight and visceral adiposity at the same time as maintaining normoleptinemia, and induced hepatic and pancreatic lipid accumulation, hyperinsulinemia, impaired glucose tolerance and hyperglucagonemia. These alterations were accompanied by hepatic glucagon resistance, as indicated by attenuated gluconeogenic gene induction and reduced CREB phosphorylation following *in vivo* glucagon stimulation. SLA‐infused rats also exhibited elevated circulating total and branched‐chain amino acids, reduced hepatic branched‐chain α‐ketoacid dehydrogenase activity and increased fibroblast growth factor 21 levels, consistent with disrupted glucagon–amino acid signalling. Together, these findings indicate that impaired central leptin signalling induces co‐ordinated endocrine and metabolic disturbances in the absence of obesity, supporting a role for altered central neuroendocrine regulation in the early development of metabolic dysfunction.

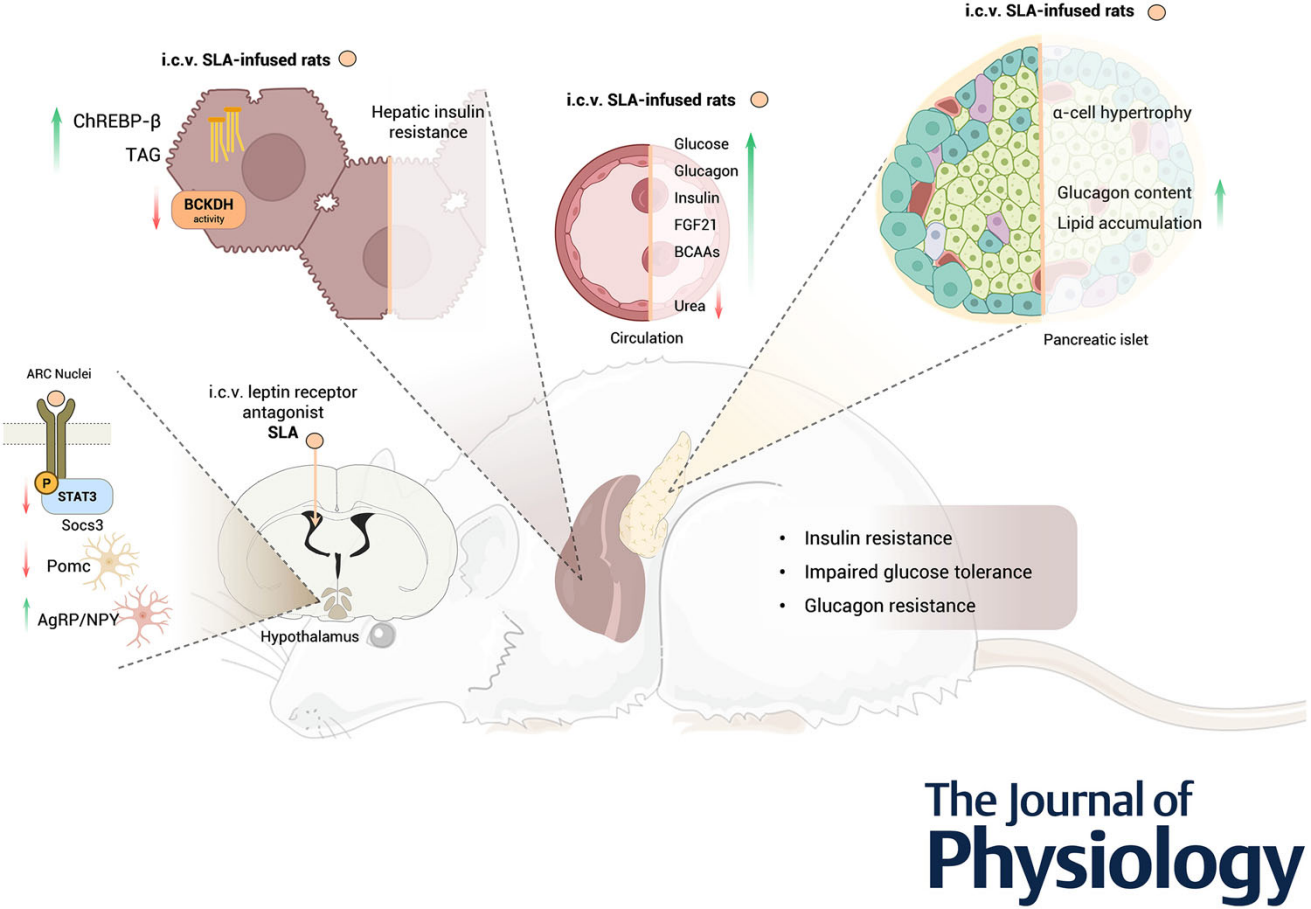

**Key points:**

- Chronic attenuation of brain leptin signalling is associated with early metabolic dysfunction in lean rats.
- Central leptin disruption leads to hyperglucagonemia, hepatic glucagon resistance and altered amino acid metabolism.
- Elevated fibroblast growth factor 21 levels and impaired branched‐chain amino acid catabolism reflect early liver–α‐cell axis dysregulation.
- This model provides insight into neuroendocrine drivers of non‐obese prediabetes.

## Graphical abstract

**Abstract figure legend** Central leptin signalling blockade induces early metabolic dysfunction. Intracerebroventricular infusion of a leptin receptor antagonist (SLA) in rats disrupts hypothalamic leptin signalling, leading to reduced signal transducer and activator of transcription 3 (STAT3) activation, decreased *Socs3* and *Pomc* expression, and increased *Agrp/Npy* activity in arcuate nucleus neurons. This central leptin signalling deficiency triggers systemic metabolic alterations. In the liver, increased ChREBP‐β expression and triacylglycerol (TAG) accumulation occur alongside reduced branched‐chain α‐ketoacid dehydrogenase (BCKDH) activity and the development of hepatic insulin resistance. Circulating metabolic markers are altered, including increased glucose, glucagon, insulin, fibroblast growth factor 21 (FGF21) and branched‐chain amino acids (BCAAs), with reduced urea levels. In pancreatic islets, α‐cell hypertrophy is observed together with increased glucagon content and lipid accumulation. Collectively, these changes produce a phenotype characterized by insulin resistance, impaired glucose tolerance and glucagon resistance, supporting this model as an early stage of metabolic dysfunction induced by impaired central leptin signalling.

## Introduction

Leptin is a key adipokine that communicates the status of peripheral energy stores to the brain, co‐ordinating appetite, energy expenditure and glucose metabolism. Loss‐of‐function mutations in leptin or its receptor cause severe obesity and metabolic dysfunction, whereas most individuals with common obesity exhibit hyperleptinemia accompanied by central leptin resistance (Ahima et al., 1996; Myers et al., 2009; Pelleymounter et al., 1995). Although these conditions highlight the importance of leptin signalling in energy balance, they predominantly reflect advanced stages of metabolic disease.

Through autonomic and neuroendocrine outputs, central leptin signalling exerts broad control over peripheral metabolism, regulating hepatic lipid handling, pancreatic hormone secretion and glucose homeostasis. Leptin also modulates sympathetic outflow to adipose tissues, thereby influencing lipid metabolism, thermogenesis and adipokine release (Buijs et al., 2001; Croizier et al., 2016; Harris et al., 2007; Myers et al., 2009). Disruption of this integrative regulatory network, through autonomic denervation or impaired leptin signalling, can promote lipid accumulation and inflammation in adipose depots, thereby exacerbating metabolic stress in the liver and other peripheral tissues (Bonzón‐Kulichenko et al., 2009, 2018; Gallardo et al., 2007; Hackl et al., 2019; Metz et al., 2022). Collectively, these observations suggest that alterations in central leptin action may contribute to metabolic dysfunction not only in overt obesity, but also during earlier stages of disease progression.

Glucagon is a key metabolic hormone regulating hepatic lipid, amino acid and glucose metabolism. In metabolic disease, hyperglucagonemia is frequently associated with hyperglycaemia, hyperinsulinemia and insulin resistance in both obesity and type 2 diabetes (Grøndahl et al., 2022; Reaven et al., 1987). Administration of exogenous leptin reduces circulating glucagon levels in leptin‐deficient *ob/ob* mice and in streptozotocin‐induced diabetic models characterized by marked hyperglucagonemia (Della‐Fera et al., 2005; Denroche et al., 2011; Dubuc et al., 1977; Yu et al., 2008). These findings indicate that leptin signalling can restrain glucagon secretion under conditions of obesity or insulin deficiency; however, it remains unclear whether endogenous leptin action within the brain contributes to the regulation of glucagon secretion and circulating glucagon levels in non‐obese states.

Emerging evidence further suggests that hepatic steatosis is associated with elevated plasma glucagon concentrations and the development of hepatic glucagon resistance (Wewer Albrechtsen et al., 2018; Winther‐Sørensen et al., 2020). In the fasting state, hyperglucagonemia may exacerbate hepatic glucose production, contributing to hyperglycaemia, whereas, in the fed state, impaired hepatic responsiveness to glucagon may partially buffer excessive glucose output, thereby influencing glucose tolerance (Bozadjieva Kramer et al., 2021). Together, these observations underscore the complexity of glucagon regulation across nutritional states and highlight the need to better understand how central neuroendocrine signals, including leptin, integrate with liver–α‐cell communication to shape glucagon action during early metabolic dysfunction.

Although the metabolic consequences of leptin deficiency and leptin resistance have been extensively characterized in obese models and are closely associated with elevated circulating leptin levels (Hu et al., 2025; Myers et al., 2010), the specific impact of impaired central leptin signalling in lean, normoleptinemic animals remains incompletely understood. Clarifying whether reduced brain leptin action contributes to early features of metabolic dysfunction, including insulin resistance, dysregulated glucagon secretion and action, and ectopic lipid accumulation, would advance our understanding of the role of the CNS in the early stages of diabetes pathogenesis, before overt obesity develops. Peripheral administration of a mouse‐specific leptin receptor antagonist (SMLA) has been shown to induce insulin resistance (Gertler & Elinav, 2014; Shpilman et al., 2011; Solomon et al., 2014). Extending these observations, we recently demonstrated that chronic central infusion of a rat‐specific superactive leptin receptor antagonist (SLA) disrupts central pathways controlling energy balance, resulting in modest increases in body weight (BW) and adiposity (Mazuecos et al., 2024). In the present study, we further examined whether reduced brain leptin action is associated with early metabolic dysfunction, including insulin resistance, dysregulated glucagon secretion and action, and ectopic lipid accumulation, under non‐obese conditions.

## Methods

### Ethical approval

Animals were handled in accordance with European Union laws (2010/63/EU) and following Spanish regulations (RD 53/2013) for the use of laboratory animals. All experimental procedures with animals were approved by the Institutional Committee for Ethical Animal Care CEEA/UCLM/JCCM (PR‐9‐2020 and PR‐16‐2024). This work complies with the ethical principles under which *The Journal of Physiology* operates and was conducted in accordance with the ARRIVE guidelines for reporting *in vivo* experiments. Sample size calculation was conducted using the GRANMO Sample Size and Power Calculator, version 7.12 (Municipal Institute of Medical Research of Barcelona, Barcelona, Spain), accepting an alpha risk of 0.05 and a beta risk of 0.2 (power = 80%) in a two‐sided test.

### Experimental model

**Figure 1:**
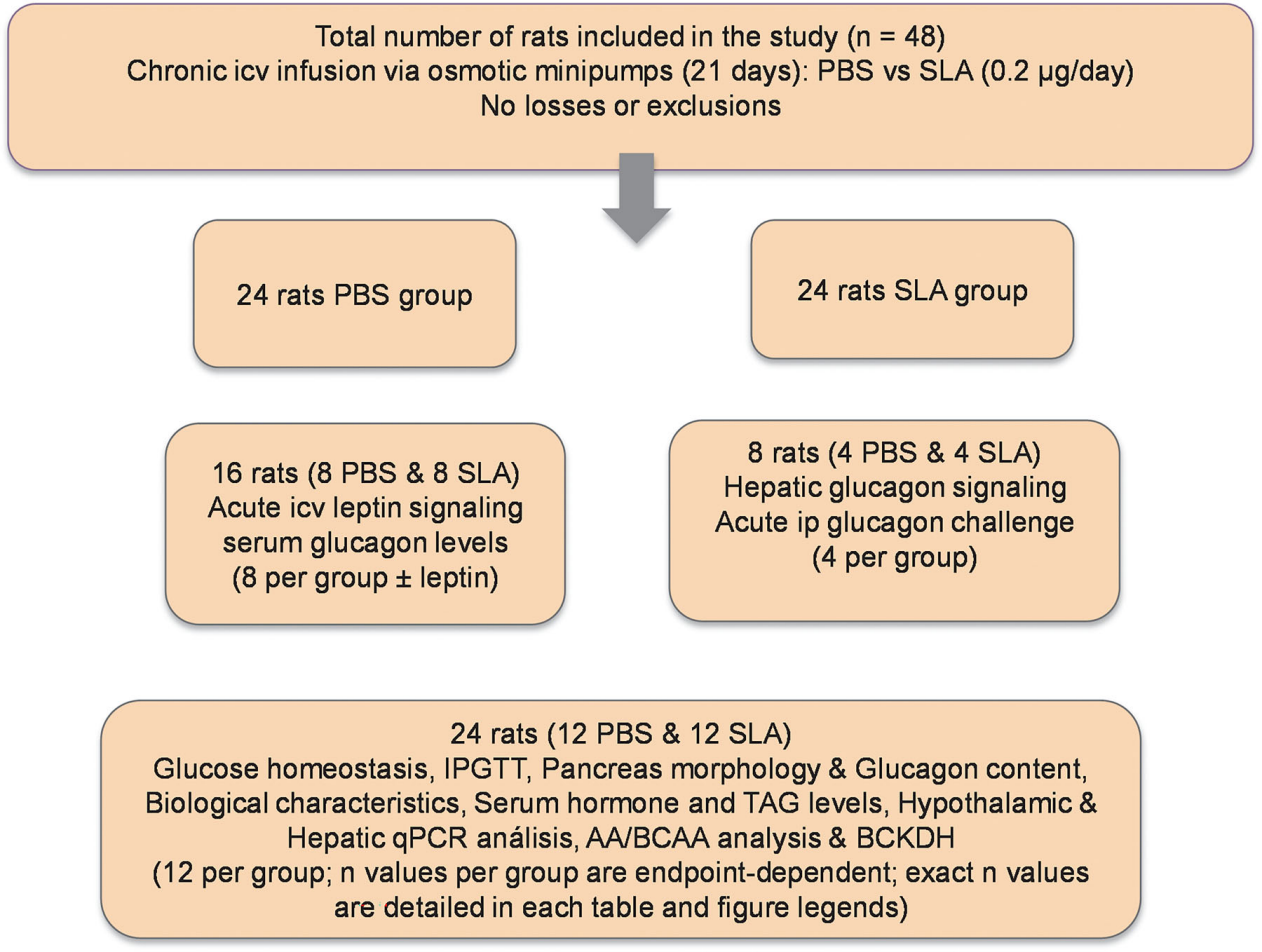
Overview of experimental design and animal includes in the study Initial group sizes were PBS (*n* = 24) and SLA (*n* = 24), with no losses or exclusions. Subsets of animals were allocated to individual analyses; exact *n* values are detailed where appropriate.

Three‐month‐old male Wistar rat, from the National Paraplegic Hospital (Toledo, Spain), were maintained under a 12:12 h light/dark photocycle (lights in 08.00 h) at 20–25° temperature and 50–55% relative humidity, Animals were fed *ad libitum* with a standard chow diet (Chow 2014; Harlan Teklad, Madison, WI, USA) and provided free access to water. Before surgery, animals were randomly allocated to predefined experimental cohorts receiving either phosphate‐buffered saline (PBS) or SLA treatment. After all surgeries, rats were randomly housed individually for daily control of food intake (FI) and BW, thereby reducing variability in weight, serum levels of hormones and metabolites depending on the amount of food eaten, and routinely monitored for any signs of stress or discomfort. Core body temperature (rectal temperature) was measured with a rectal thermometer (#8851 K.J.T. Type; Bioseb, Vitrolles, France). An experimental overview and number of animals included in the study is shown in Fig. 1.

### *In* v*ivo* treatment studies

#### Study 1: chronic i.c.v. infusion of leptin receptor antagonist (SLA)

Intracerebroventricular infusion of leptin receptor antagonist was performed as described previously (Mazuecos et al., 2024). Animals were anaesthetized in an induction chamber by inhalation of a mixture of 4% isoflurane (Pharmacia‐Upjohn, Barcelona, Spain) in 96% O_2_ at a flow rate 0.8 L min^−1^. For surgeries, animals were placed in a stereotaxic frame (David Kopf, Tujunga, CA, USA) on a thermal blanket underneath and maintained under anaesthesia with 1.5% isoflurane in 98.5% O_2_ at a flow rate of 0.4 L min^−1^ delivered through a gas anaesthetic mask during surgery. Meloxicam was injected s.c. (2 mg kg^−1^; #04400015; Metacam 5 mg mL^−1^; Boehringer Ingelheim, Sant Cugat del Vallès, Spain) as an analgesic prior to surgery and every 24 h for 72 h post‐surgery. An opening in the skull was made with a dental drill at –1.6 mm lateral to the midline and 0.8 mm anterior to the bregma. Then a cannula connected to an osmotic minipump (Alzet, Palo Alto, CA, USA) was implanted in the left ventricle for chronic infusion for 21 days of SLA (0.2 µg day^−1^) (rat‐specific super active leptin receptor antagonist, D23L/l39A/D40A/F41A, MBS400108; MyBiosource, San Diego, CA, USA), or its vehicle (PBS). Osmotic minipumps, with a release rate of 0.25 µL h^−1^, were filled with 0.0332 µg µL^−1^ SLA (treated rats) or PBS (control rats). The total number of rats included in this study was 48 (*n* = 24 PBS and *n* = 24 SLA) and killed after 21 days of treatment.

#### Study 2: i.c.v. treatment with SLA or PBS for 21 days followed by an acute central leptin infusion

Additionally, on a subset of each group (*n = *8 PBS and *n* = 8 SLA) we performed an acute brain leptin signalling stimulation at the end of the 21‐day infusion with PBS or SLA. To this end, a guide cannula was pre‐implanted, at day 0, in the contralateral ventricle for the posterior acute infusion of leptin or PBS. A dummy cannula was inserted into the guide cannula and secured in place by screwing it onto the guide to prevent contamination. After 21 days of chronic i.c.v. SLA or PBS infusion, overnight fasted (16 h) rats were transiently anaesthetized as above, with 4%/1.5% isoflurane in 96–98.5% O_2_ inhalation (induction and maintenance, respectively) and acutely treated for 2 min with 2 µL of either rat leptin (20 ng) (Sigma‐Aldrich, St Louis, MO, USA) or its vehicle (PBS). This leptin dose was selected to induce a robust yet submaximal signal transducer and activator of transcription 3 (STAT3) phosphorylation response at the same time as preserving dynamic range for detecting altered leptin responsiveness. Rats were allowed to recover and, 30 min later, were placed in a chamber and killed by 100% CO_2_ inhalation. Death was confirmed by the absence of vital signs and lack of reflex responses, followed by decapitation. Blood was removed and centrifuged (2000 ***g*** for 15 min), and serum was recovered and frozen in liquid nitrogen for storage at –80°C until use. The whole hypothalamus, liver and epididymal and retroperitoneal fat pads were carefully dissected, weighed and stored at –80°C until use. The total number of rats included in this study was 16 (eight rats per group acutely stimulated for 30 min with leptin (*n* = 4) or PBS infused (*n* = 4) on day 21).

#### Study 3: hepatic glucagon signalling

After 21 days of i.c.v. PBS or SLA infusion, on a subset of each group (*n = *4 PBS and *n* = 4 SLA) of overnight fasted (16 h) rats were weighed and injected i.p. for 30 min with 100 µg of human glucagon kg^−1^ BW to analyse the liver glucagon signalling activation by measuring canonical phosphorylation of the transcription factor CREB (i.e. cAMP response element‐binding protein). The glucagon dose was selected to achieve maximal CREB phosphorylation as reported previously (Bozadjieva Kramer et al., 2021; Merino et al., 2022). Blood samples were obtained from the tail tip at 0, 5, 15 and 30 min after i.p. glucagon administration, and glucose levels were determined using an Accutrend Glucose Analyzer (Roche Diagnostics Corp., Indianapolis, IN, USA). The overall change in glucose during the glucagon challenge assay was calculated as the area under the curve (AUC) using Prism, version 10.0.0 (GraphPad Software Inc., San Diego, CA, USA). After the treatment ended, rats were killed as described above. Livers were dissected, immediately frozen in liquid nitrogen and subsequently stored at −80°C for the analysis of hepatic glucagon signalling. The total number of rats included in this study was eight (four rats per group acutely stimulated for 30 min with glucagon on day 21).

Visceral adiposity is represented as the summatory in grams of epididymal and retroperitoneal fat tissue weights, divided by BW × 100. Interscapular brown adipose tissue mass (g) was normalized to BW and expressed as (tissue mass/BW) × 100. Obesity Lee index was calculated as cube root of BW in grams divided by naso‐anal length in millimetres × 10^4^ (Bernardis et al., 1982).

### Serum hormone and metabolite analysis

Serum hormone and metabolite levels were measured using commercially available rat‐specific enzyme‐linked immunoassay kits (ELISA) kits or colorimetric kits, respectively, in accordanvce with the manufacturer's instructions as described previously (Fernández et al., 2019; López et al., 2018). Serum insulin and glucagon levels were determined using rat ELISA assays (#10‐1250‐01; RRID:AB_2811229 for insulin and #10‐1281‐01; RRID:AB_2783839 for glucagon; Mercodia AB, Uppsala, Sweden). Corticosterone was determined by a direct sandwich ELISA kit (#ab108821; RRID AB_2889904; Abcam, Cambridge, UK). FGF21 was determined using a mouse/rat ELISA assay (#RD291108200R; RRID:AB_2909467; BioVendor, Heidelberg, Germany). Serum leptin, C‐peptide, glucose‐dependent insulinotropic polypeptide (GIP) and peptide YY (PYY) levels were measured using a multiplexed rat metabolic hormone magnetic bead immunoassay panel (RMHMAG‐84K) from Merk‐Millipore (Darmstadt, Germany). Beads were analysed in the Bio‐Plex suspension array system 200 (Bio‐Rad Laboratories, Madrid, Spain). Raw data (mean fluorescence intensity) were analysed using Bio‐Plex Manager Software 6.2. (Bio‐Rad Laboratories). Serum triglycerides (TAG) were measured using an enzymatic kit (#23528; Biosytems, Barcelona, Spain). β‐Hydroxybutyrate (β‐OHB) and urea levels in serum were measured with colorimetric assay kits (β‐OHB, #700 190; Cayman Chemical, Ann Arbor, MI, USA) and (urea, ab83362; Abcam). Hepatic neutral lipid extraction and hepatic TAG content were assessed as reported previously (Gallardo et al., 2007). Serum total and branched‐chain amino acid (BCAA) levels were measured with l‐amino acids (MAK002‐01) or BCAA (MAK003‐01) quantitation kits (Sigma‐Aldrich), respectively.

Individual amino acid levels in serum were quantified by RP‐HPLC using a diethylethoxymethylenemalonate (DEEMM) (D94208; Sigma‐Aldrich) derivatization method, as described previously (Poveda et al., 2017). A mixture of 1 mL of the sample, 1.75 ml of 1 mol L^−1^ borate buffer pH 9.0, 750 µL of methanol, 20 µLml of internal standard (l‐2‐aminoadipic acid, (freshly) 1 mg/ mlL^−1^ in 0.1 mol/ lL^−1^ HCl) (Sigma‐Aldrich) and 30 (lΛ of DEEMM were incubated at 30°C in an ultrasonic bath for 30 min. The samples were then heated at 70°C for 2 h to allow the complete degradation of excess DEEMM and reagent by‐products. After derivatization, the samples were filtered through regenerated cellulose esters 0.2 µm membranes coupled to a syringe into conical vials. The samples were derivatized in duplicate just before injection into the chromatograph system. The analysis was performed using an Agilent 1200 HPLC (Agilent, Santa Clara, Ca, USA), equipped with a Zorbax Eclipse XDB C18 column particle size 5 mm (250  ×  4.6 mm) (Agilent), an Agilent guard cartridge C18 particle size 5 mm (12.5 mm × 4.6 mm) (Agilent) and a photodiode array detector. The target compounds were identified by their retention times and their spectral characteristics at 280 nm and were quantified using the internal standard method. The glucagon‐alanine index (the product of serum fasting levels of glucagon (pmol/ lL^−1^) and alanine (µmol/ lL^−1^) was conducted as described (Wewer Albrechtsen et al., 2018).

### Intraperitoneal glucose tolerance test (IPGTT)

To determine the effect of SLA infusion on glucose homeostasis, we performed an IPGTT on two different days of treatment, on days 14 and 21 of treatment, performed as previously described (Bonzón‐Kulichenko et al., 2011). First, on day 14, central‐infused PBS or SLA rats were fasted overnight for 16 h, weighed and then injected i.p. with 2 g of glucose kg^−1^ BW. Blood samples were obtained from the tail tip at 0, 15, 30, 60 and 120 min after glucose administration, and glucose levels were determined using an Accutrend Glucose Analyzer (Roche Diagnostics Corp., Indianapolis, IN, USA). After the IPGTT, the rats were allowed to recover and then killed on day 21 at the end of treatment. Second, a separate subset of PBS‐ or SLA‐infused rats underwent an IPGTT on day 21 as above, after which they were killed. Overall changes in glucose, and insulin during the IPGTT were calculated as the AUC using Prism, version 10.0.0 (GraphPad Software Inc.).

### Pancreatic glucagon quantification

To determine pancreas glucagon content, after dissection, pancreata were weighed and incubated overnight in an acid–ethanol solution (1.5% v/v HCl in EtOH) at −20°C. After homogenization, samples were centrifuged at 12,000 ×  *g* for 10 min at 4°C. Glucagon levels were measured in the supernatant1 fraction, diluted 1:500, by an ELISA glucagon assay.

### Immunohistochemistry and pancreatic fat accumulation

At the end of i.c.v. treatment, overnight fasted (16 h) rats were killed, then their pancreata were dissected, weighed, fixed in 10% neutral buffer formalin overnight at 4°C, and embedded into paraffin blocks. Pancreas histomorphometry was performed on paraffin 5 µm sections mounted on glass slides, which were incubated with anti‐insulin or anti‐glucagon antibodies and counterstained with haematoxylin, as described previously (Fernández‐Díaz et al., 2019; Merino et al., 2022). Staining was performed in two sections per rat pancreas spaced at least 200 µm apart. To analyse pancreas histomorphometry, sections were incubated with guinea‐pig anti‐insulin polyclonal antibody (dilution 1:100; #ab7842, RRID:AB_306130) for β‐cell area or with mouse anti‐glucagon monoclonal antibody (dilution 1:500; ab#10988, RRID:AB_297642; both from Abcam) for α‐cell area, washed, and then incubated with horseradish peroxidase (HRP)‐conjugated secondary antibodies, donkey anti‐guinea‐pig IgG HRP (dilution 1:100; #706‐036‐148, RRID:AB_2 340448; Jackson Labs, West Grove, PA, USA) or rabbit anti‐mouse IgG HRP (dilution 1:5000; #AQ160P, RRID:AB_92795; Millipore, Burlington, MA, USA). Finally, sections were counterstained with haematoxylin. To determinate β‐cell and alpha α‐cell mass, insulin or glucagon staining was performed by one researcher who captured images at the microscope and named them ‘blind’; these pictures were then blindly counted by a different researcher.

To evaluate the intrapancreatic adipocyte infiltration, the adipocyte area was quantified on cryostat 25 µm sections of frozen pancreas after haematoxylins and eosin and Oil Red O staining. At the end of icv treatment, overnight fasted (16 h) rats were perfused with 4% paraformaldehyde. Pancreas were removed and placed in 4% paraformaldehyde and kept overnight at 4°C. Once fixed, pancreatas were cryoprotected using 30% sucrose in PBS at 4°C. Subsequently, Tissue‐Tek OCT™ (Sakura, Torrance, CA, USA) was used to embed and freeze the pancreas at –80°C. Staining was performed in two sections per rat pancreas, spaced at least 200 µm apart. The adipocyte area relative to pancreatic tissue area was quantified from images of histological sections using Image J (NIH, Bethesda, MD, USA).

### Immunoblotting

For this, 100 mg of frozen liver tissue or the entire hypothalamus were homogenized in RIPA buffer (R0278; Sigma‐Aldrich) with 2 mm Na3VO4, 10 mg/ mlL^−1^ leupeptin, 10 mg/ mlL^−1^ aprotinin, 10 mg/ mlL^−1^ and pepstatin using a manual Dounce homogenizer. The homogenates were centrifugated at 4500× ***g*** for 15 min at 4°C, and the supernatants were considered as total protein extract. All extracts were stored at ‐80°C until use. Bradford protein assay was used for total protein quantification (500‐006; Bio‐Rad, Hercules, CA, USA). About Approximately 30 µg of protein from hypothalamus and liver total extracts were separated under reducing conditions in 7.5% SDS‐PAGE. Samples were previously boiled at 90°C. Proteins were transferred to nitrocellulose sheets (0.2 µm) (Bio‐Rad Laboratories) and routinely stained with Ponceau Red to ensure protein loading and transfer efficiency prior to immunodetection. After destaining, membranes were incubated overnight at 4° C with the corresponding primary antibody, followed by incubation at room temperature for 30 min with a secondary antibody conjugated with HRP. Primary monoclonal antibodies used in WB analysis were mouse anti‐STAT3 (dilution 1:1000; #9139, RRID:AB_331757), mouse anti‐pY705‐STAT3 (dilution 1:500; #9138, RRID:AB_331262) rabbit anti‐BCKDH‐E1a (dilution 1:1000; #90198, RRID:AB_2 800155), rabbit anti‐pS293‐BCKDH‐E1a (dilution 1:1000; #40368, RRID:AB_40368), rabbit anti‐CREB (dilution 1:1000; #9197, RRID:AB_331277), rabbit anti‐pS133‐CREB (dilution 1:1000; #9198, RRID:AB_2 561044) from Cell Signaling Technology, MA, USA, as well as mouse anti‐β‐actin (dilution 1:1000; #ab8226, RRID:AB_306371; Abcam). The secondary antibody used were goat anti‐rabbit IgG conjugated with HRP (dilution 1:4000; #1721019, RRID:AB_11125143) and goat anti‐mouse IgG conjugated with HRP (dilution 1:4000; #1706516, RRID:AB_2 921252). Samples from rats infused with vehicle (PBS) or SLA in all experimental conditions were run on the same gel to allow a direct comparison and routinely checked with loading controls. Blots were repeated two times to assure the reproducibility of the results. The immunocomplexes formed were visualized using the ECL Western‐blotting detection kit (Amersham Biosciences, Inc., Piscataway, NJ, USA) and the images were subjected to a densitometric analysis with a G‐Box Densitometer (Syngene, Cambridge, UK), and bands were quantified by scanning densitometry with the exposure in the linear range using Gene Tools software (Syngene, Cambridge, UK). The densitometric values of pY705‐STAT3, pS293‐BCKDH‐E1a and pS133‐CREB were normalized with the densitometric values of the corresponding amount of protein mass in the same sample. Data were expressed as a ratio of pY705‐STAT3/STAT3, pS293‐BCKDH‐E1a/BCKDH‐E1a and pS133‐CREB/CREB. β‐actin was used as a control for protein loading of the total tissue.

### Gene expression analysis

Total RNA from the whole hypothalamus was obtained using RNeasy Lipid Tissue Mini Kit (#1023539) or with Qiazol (#79306) for liver tissue; both from (Qiagen, Hilden, Germany), in accordance with the manufacturer's instructions. Complementary DNA (cDNA) was synthesized from 1 µg of DNase‐treated RNA. Real‐time quantitative PCR (qPCR) was performed by using the ABI PRISM 7500 Fast Sequence Detection System instrument and software (Applied Biosystems, Foster City, CA, USA). Relative quantification of target cDNA in each sample was performed from 10 ng of cDNA in TaqMan One‐Step real‐time PCR Master Mix, using Pre‐Developed TaqMan Assay Reagents (Applied Biosystems) for *Acc‐α*, *Acly*, *Ccl5*, *Cd68*, *Dgat2*, *Fasn*, *Glu6Pase*, *Gk*, *Glut2*, *Hmgcs2*, *Htgl*, *Il‐1β*, *Irs‐2*, *Mttp*, *Pck1*, *Pomc*, *PPARα*, *PPARγ*, *Scd1*, *Socs3* and *Tnf‐α* with FAM and *18S rRNA* with VIC as a real time reporter used as a control to normalize gene expression. Pre‐developed TaqMan probes for real‐time PCR are listed in the Appendix (Table A1). For *Agrp*, *Bdk*, *ChREBP‐α*, *ChREBP‐β*, *Cps1*, *Crtc2*, *E1‐α*, *E1‐β*, *E2*, *Got1*, *Lat3*, *Ppm1k* and *18S rRNA* as a housekeeper to normalize gene expression, we used the SYBR‐Green One‐Step real‐time PCR Master Mix with the primer sequences for amplifying the target genes (Merck Life Science, Madrid, Spain) as listed in the Appendix (Table A2). Primers were validated for specificity using melting curve analysis. Relative gene expression levels were quantified using the ΔΔCT method, with fold changes calculated between experimental and control groups. Expression values were normalized to the mean expression of the housekeeping gene *18S rRNA*, which remained stable across all groups.

### Statistical analysis

All data are expressed as the mean ± SD for the indicated number of observations. Sample sizes were consistent with those used in our previous studies with this experimental model and in studies conducted by other groups (Gallardo et al., 2007; Hackl et al., 2019; Mazuecos et al., 2024; Metz et al., 2022; Winther‐Sørensen et al., 2020). Normal distribution of the data was checked with the Shapiro–Wilk test. When the distribution of data was normal, differences between experimental groups were determined using unpaired two‐tailed Student's *t* test followed by Welch's correction, as appropriate, or two‐way ANOVA followed by Holm–Šidák multiple‐comparison testing. Non‐parametric data were analysed using the Mann–Whitney test. Statistical analyses were performed using Prism, version 10.0.0 (GraphPad Software Inc.). The number of animals included in each experimental analysis is reported where appropriate. *P* < 0.05 was considered statistically significant.

## Results

### Central SLA infusion attenuates hypothalamic leptin receptor signalling

**Figure 2:**
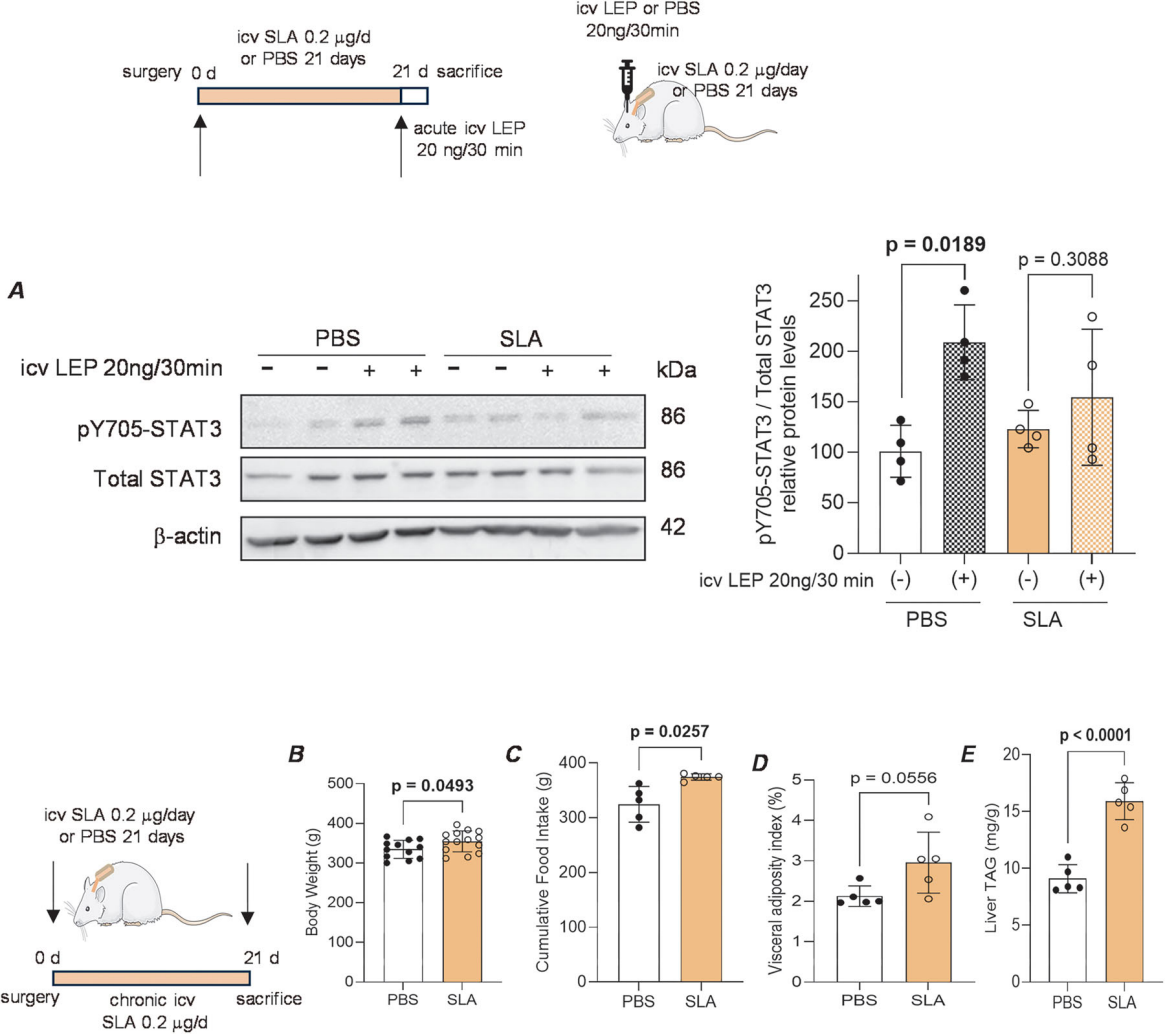
Central leptin receptor antagonism blunts STAT3 signalling and is associated with early adiposity changes under non‐obese conditions *A*, quantification of hypothalamic pTyr705‐STAT3 and total STAT3 by immunoblot following acute leptin or vehicle administration after 21 days of chronic PBS or SLA treatment. *B* and *C*, daily body weight (*B*) and daily food intake (*C*) monitored throughout the 21‐day infusion period. *D*, visceral adiposity index (%). *E*, hepatic triacylglycerol (TAG) content. *n* = 4 per group (*A*); *n* = 12–14 per group (*B* and *C*); *n* = 5 per group (*C*–*E*). Data are presented as the mean ± SD. *P* values in bold indicate statistical significance.

**Figure A1:**
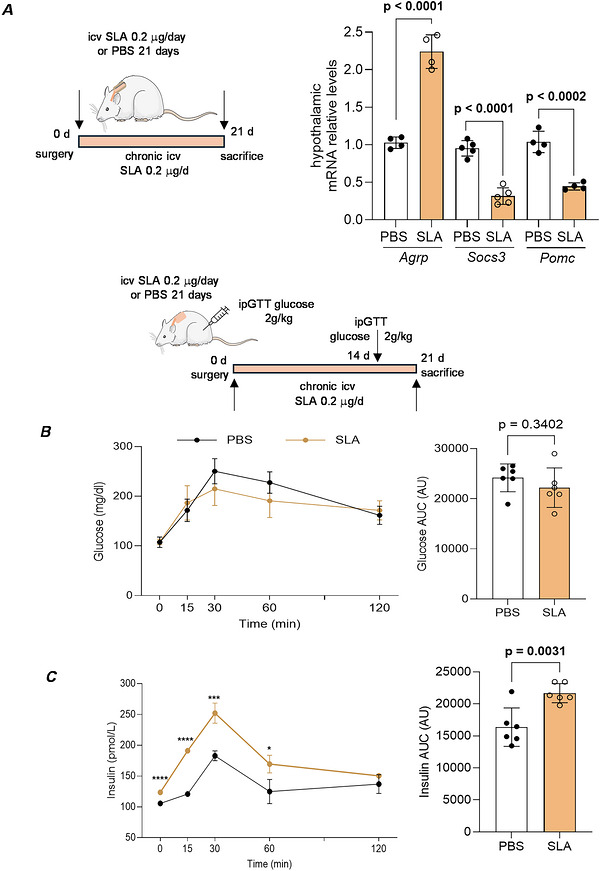
Chronic hypothalamic brain leptin signalling deficiency alters the expression of genes involved in food intake and glucose homeostasis *A*, hypothalamic mRNA expression levels of *Socs3*, *Agrp* and *Pomc* after 21 days of central PBS or SLA (0.2 µg day^−1^) infusion (*n* = 4–5 per group). *B*, fasting (16 h) blood glucose levels during the i.p. glucose tolerance test (IPGTT) and corresponding area under the curve (AUC) after 14 days of central PBS or SLA infusion (*n* = 6 per group). *C*, fasting (16 h) serum insulin levels during the IPGTT and corresponding AUC (*n* = 6 per group). AUC, area under the curve. Data are the mean ± SD. *P* values in bold indicate statistical significance.

We first assessed whether chronic i.c.v. infusion of the selective leptin receptor antagonist (SLA) for 21 days impairs hypothalamic leptin receptor signalling by examining activation of signal transducer and activator of transcription 3 (STAT3), a key mediator of leptin's central metabolic actions (Myers et al., 2010). Acute i.c.v. leptin administration robustly increased hypothalamic STAT3 phosphorylation (pTyr705‐STAT3) by approximately two‐fold in PBS‐infused control rats, whereas SLA‐infused rats exhibited only a modest ∼1.2‐fold increase (Fig. 2*A*), indicating attenuated leptin‐induced STAT3 activation. Chronic SLA infusion was also associated with altered arcuate nucleus neuropeptide expression, including significant upregulation of *Agrp* and marked reductions in *Pomc* and *Socs3 m*RNA levels (see Appendix, Fig. A1*A*), consistent with impaired central leptin action. Accordingly, SLA‐infused rats exhibited a moderate significant increase in BW and cumulative FI compared to PBS‐infused control rats (Fig. 2*B* and *C*). Nevertheless, no significant difference in the Lee index, a commonly used measure of obesity in rats (Bernardis et al., 1982), was observed between the PBS‐ and SLA‐infused groups, indicating comparable overall adiposity (Table 1).

**Table 1:** Effects of i.c.v. SLA infusion for 21 days on the biological characteristics of the rats

	PBS	SLA	*P*	*n*
Obesity Lee index	302.6 ± 2.5	302.8 ± 3.65	*P* = 0.4995	8
iBAT mass per BW (%)	0.10 ± 0.021	0.12 ± 0.026	*P* = 0.1255	6
Core body temperature°C	33.13 ± 0.22	32.65 ± 0.12	*P* = **0.0101**	4
Fasting serum TAG (mg dL^−1^)	130.2 ± 34.39	175.8 ± 30.17	*P* = **0.0564**	5
Fasting serum leptin (ng mL^−1^)	1.67 ± 0.72	1.61± 0.42	*P* = 0.8413^#^	5
Serum corticosterone (ng mL^−1^)	92.54 ± 53.1	167.4 ± 156.4	*P* = 0.3085	6
Liver glycogen (ng g^−1^)	3.88 ± 1.29	5.40 ± 3.30	*P* = 0.2572	8
Fasting serum GIP (pg mL^−1^)	43.26 ± 10.78	26.55 ± 7.37	*P* = **0.0138**	6
Fasting serum PYY (pg mL^−1^)	18.28 ± 2.08	9.93 ± 1.19	*P* < **0.0001**	6

Values are expressed as the mean ± SD; *n* indicates the number of animals per group. *P* indicates *P* values for comparison of PBS control and SLA infused rats determined by unpaired two‐tailed Student's *t *test followed by Welch´s correction, as appropriate or by using the Mann–Whitney test. *P* values in bold indicate statistical significance. iBAT, interscapular brown adipose tissue; PBS, vehicle‐infused control rats; SLA, leptin receptor antagonist‐infused rats.

Visceral adipose tissue mass was modestly increased in SLA‐treated rats (Fig. 2*D*), accompanied by elevated hepatic triacylglycerol (TAG) content (Fig. 2*E*) and increased fasting serum TAG levels (Table 1), consistent with early adiposity changes and incipient hepatic lipid accumulation under non‐obese conditions. Although no differences in interscapular brown adipose tissue mass were observed, core body temperature was significantly decreased in SLA‐infused rats (Table 1). Notably, fasting serum leptin concentrations remained comparable between the PBS‐ and SLA‐infused groups, indicating preserved peripheral leptin levels (Table 1). In addition, SLA‐infused rats displayed no significant changes in hepatic glycogen content and circulating corticosterone levels (Table 1), suggesting that these parameters probably do not represent primary drivers of the metabolic alterations observed.

### Reduced brain leptin signalling is associated with altered glucose homeostasis and insulin sensitivity

**Figure 3:**
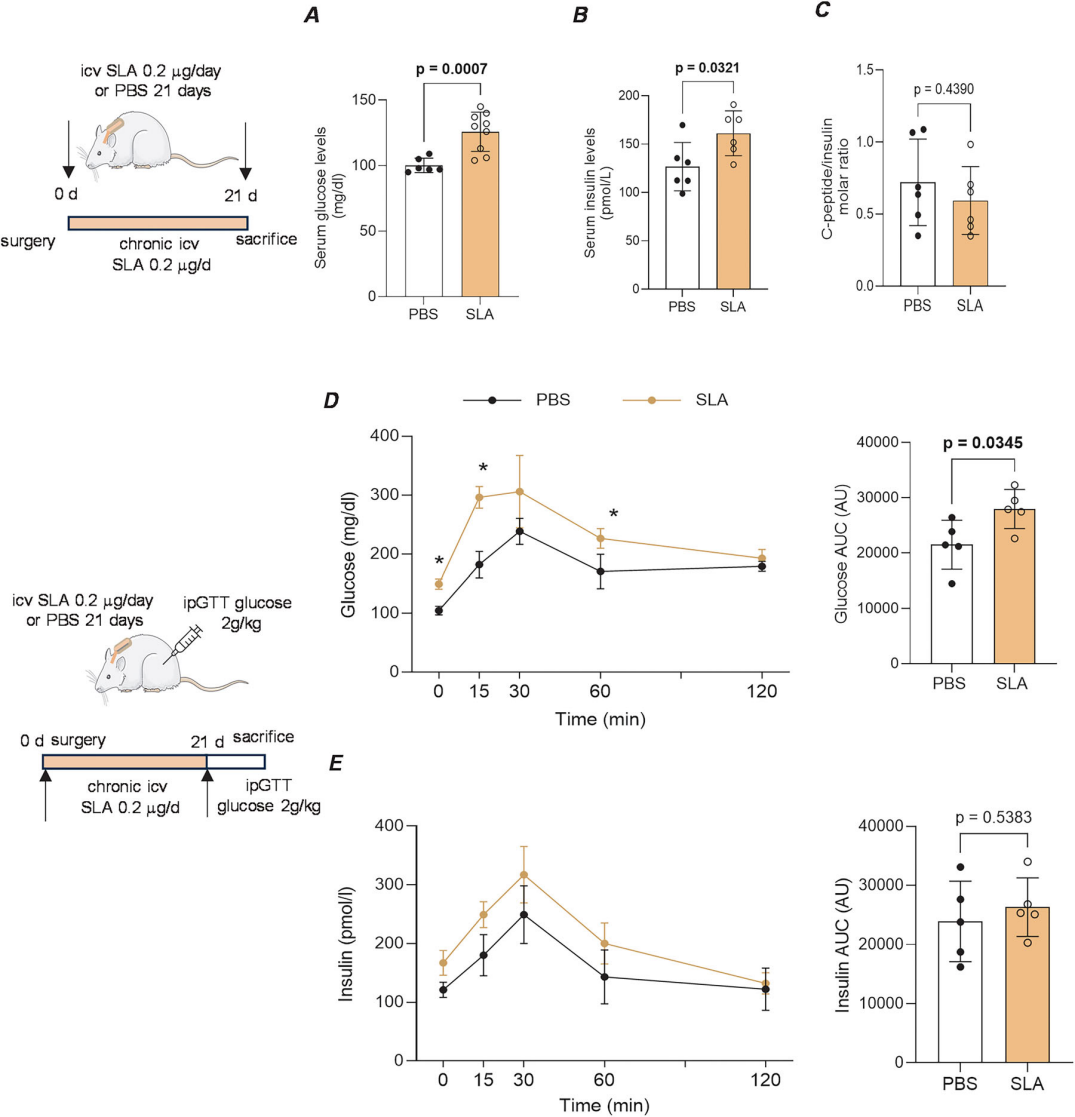
Impaired central leptin signalling is associated with altered glucose homeostasis and insulin0 resistance *A*–*C*, fasting serum glucose (*A*), insulin (*B*) and C‐peptide‐to‐insulin molar ratio (*C*) after 21 days of intracerebroventricular PBS or SLA infusion. *D* and *E*, blood glucose levels during an i.p. glucose tolerance test (IPGTT) and corresponding area under the curve (AUC) (*D*) and serum insulin levels during IPGTT and corresponding AUC (*E*). *n* = 6–9 per group (*A*); *n* = 6 per group (*B*); *n* = 6 per group (*C*); *n* = 5 per group (*D*); *n* = 5 per group (*E*). *D*, *P < 0.05. Data are presented as the mean ± SD. *P* values in bold indicate statistical significance.

At an intermediate time point (day 14), when BW had not yet increased significantly (PBS‐infused: 337.2 ± 11.6 g, *n* = 6; SLA‐infused: 335.8 ± 12.8 g, *n* = 6; *P* = 0.9400) and differences in fasting glucose levels or glucose excursion during the glucose load were minimal (see Appendix, Fig. A1*B*), the SLA‐infused rats manifest fasting hyperinsulinemia and enhanced insulin secretion during the glucose load (see Appendix, Fig. A1*C*), indicating that attenuated brain leptin signalling precedes development of obesity and insulin resistance (Münzberg et al., 2004). However, by day 21, SLA‐infused rats exhibited fasting hyperglycaemia and hyperinsulinemia (Fig. 3*A* and *B*). IPGTT revealed impaired glucose tolerance and higher circulating insulin during the glucose challenge in SLA‐infused rats, consistent with whole‐body insulin resistance. The C‐peptide/insulin ratio was not altered, suggesting no major change in insulin clearance at this stage (Fig. 3*C*).

### Central SLA infusion is associated with altered glucagon dynamics

**Figure 4:**
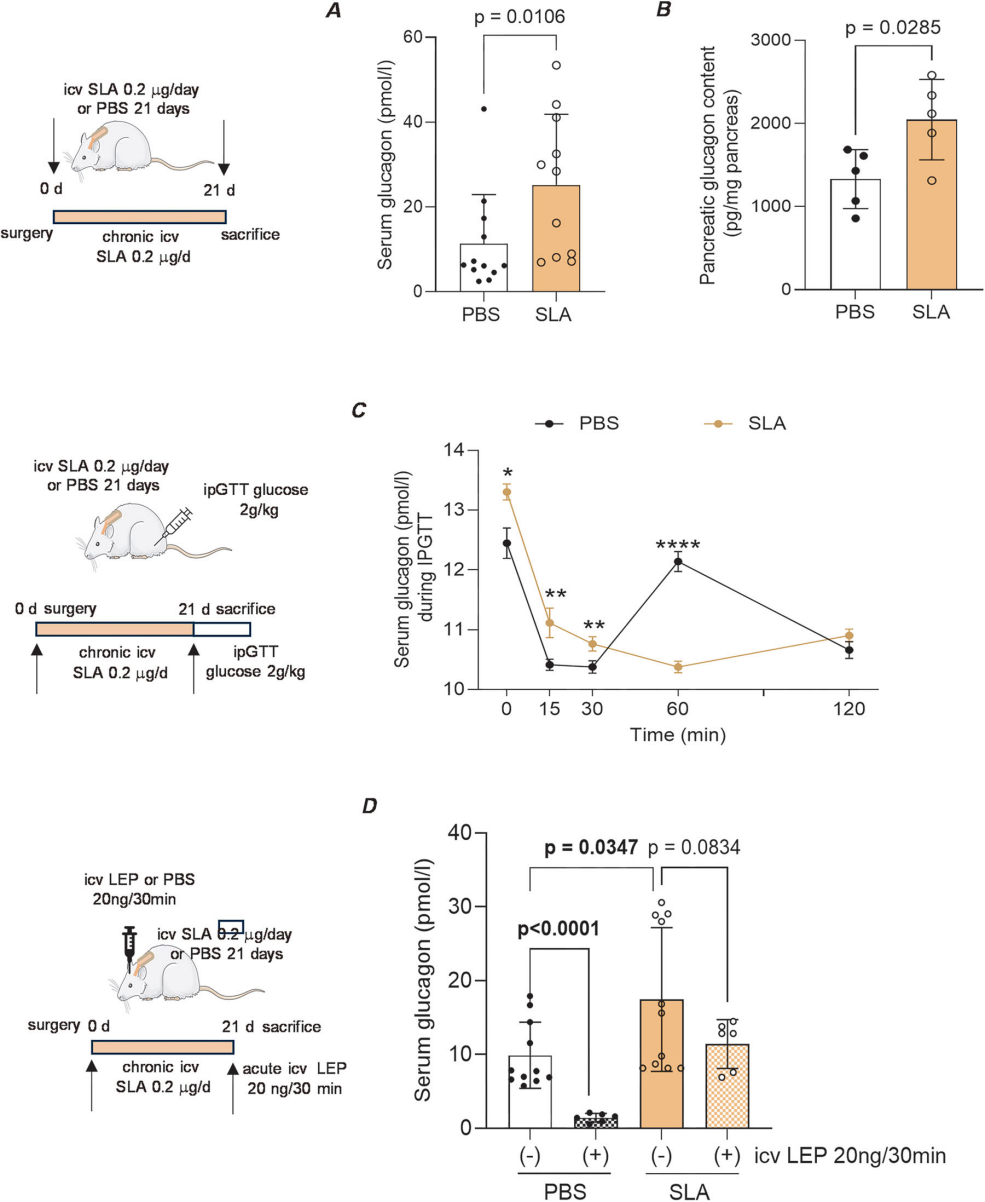
Chronic central leptin receptor antagonism is associated with altered glucagon dynamics and hepatic glucagon signalling *A*, fasting serum glucagon concentrations. *B*, fasting pancreatic glucagon content. *C*, serum glucagon levels during an i.p. glucose tolerance test (IPGTT) and corresponding area under the curve (AUC). *D*, serum glucagon levels following acute leptin or vehicle administration after chronic PBS or SLA treatment. *n* = 11–12 per group(*A*); *n* = 5 per group (*B*); *n* = 6 per group (*C*); *n* = 6–11 per group (*D*). *C*, *P < 0.05, **P < 0.01, ****P < 0.0001. Data are presented as the mean ± SD. *P* values in bold indicate statistical significance.

We next examined whether impaired central leptin signalling affects glucagon secretion and circulating glucagon levels. SLA‐infused rats exhibited fasting and post‐load hyperglucagonemia, together with increased total pancreatic glucagon content (Fig. 4*A*–*C*). During the IPGTT, the normal suppression of glucagon secretion observed in control rats was attenuated in SLA‐treated animals (Fig. 4*C*). Moreover, the temporal pattern of glucagon secretion differed between the PBS‐ and SLA‐infused group. Control rats displayed the characteristic U‐shaped glucagon response during the IPGTT, whereas this pattern was blunted in SLA‐infused rats, with reduced suppression during the first 30 min following glucose administration (Fig. 4*C*).

In addition, whereas serum glucagon levels increased between 30 and 60 min in control rats during the IPGTT, this response was not observed in SLA‐treated animals (Fig. 4*C*). Acute i.c.v. leptin administration markedly reduced circulating glucagon levels in PBS‐treated controls (∼80% reduction), whereas only a smaller, non‐significant decrease (∼37%) was observed in SLA‐infused rats (Fig. 4*D*).

### Pancreatic adipocyte infiltration accompanies α‐cell remodelling

**Figure 5:**
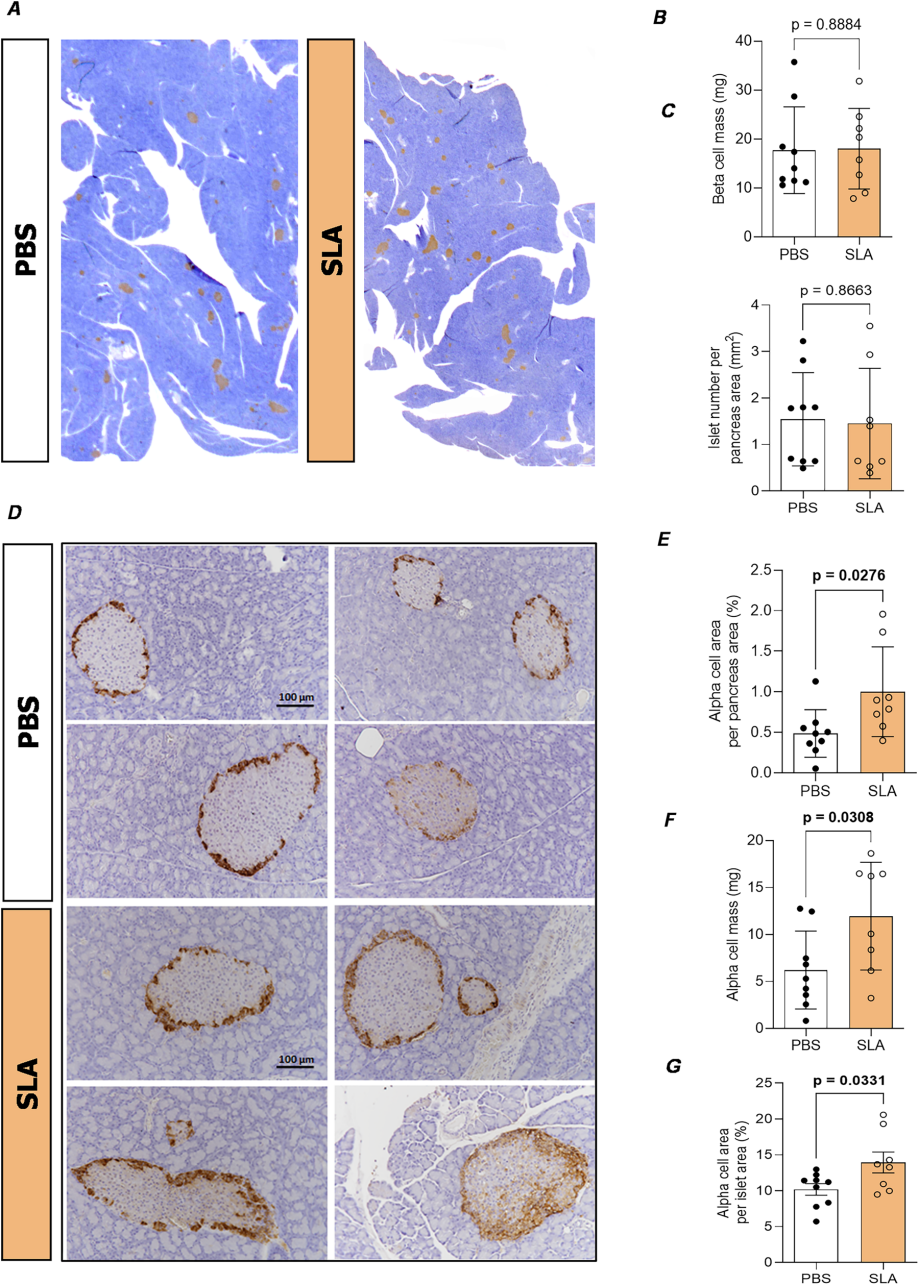
Central leptin signalling deficiency is associated with pancreatic α‐cell expansion *A*, representative immunohistochemistry for insulin in pancreatic sections from rats centrally infused with PBS or SLA for 21 days. *B* and *C*, β‐cell mass (*B*) and islet number (*C*) per pancreatic section. *D*, representative immunohistochemistry for glucagon in pancreatic sections from PBS‐ or SLA‐treated rats. *E*–*G*, α‐cell area relative to total pancreatic area (*E*), α‐cell mass (*F*) and α‐cell area relative to islet area (*G*). *n* = 9 (PBS) and *n* = 8 (SLA) (*B* and *C* and *E*‐*G*). Data are presented as the mean ± SD. *P* values in bold indicate statistical significance.

**Figure 6:**
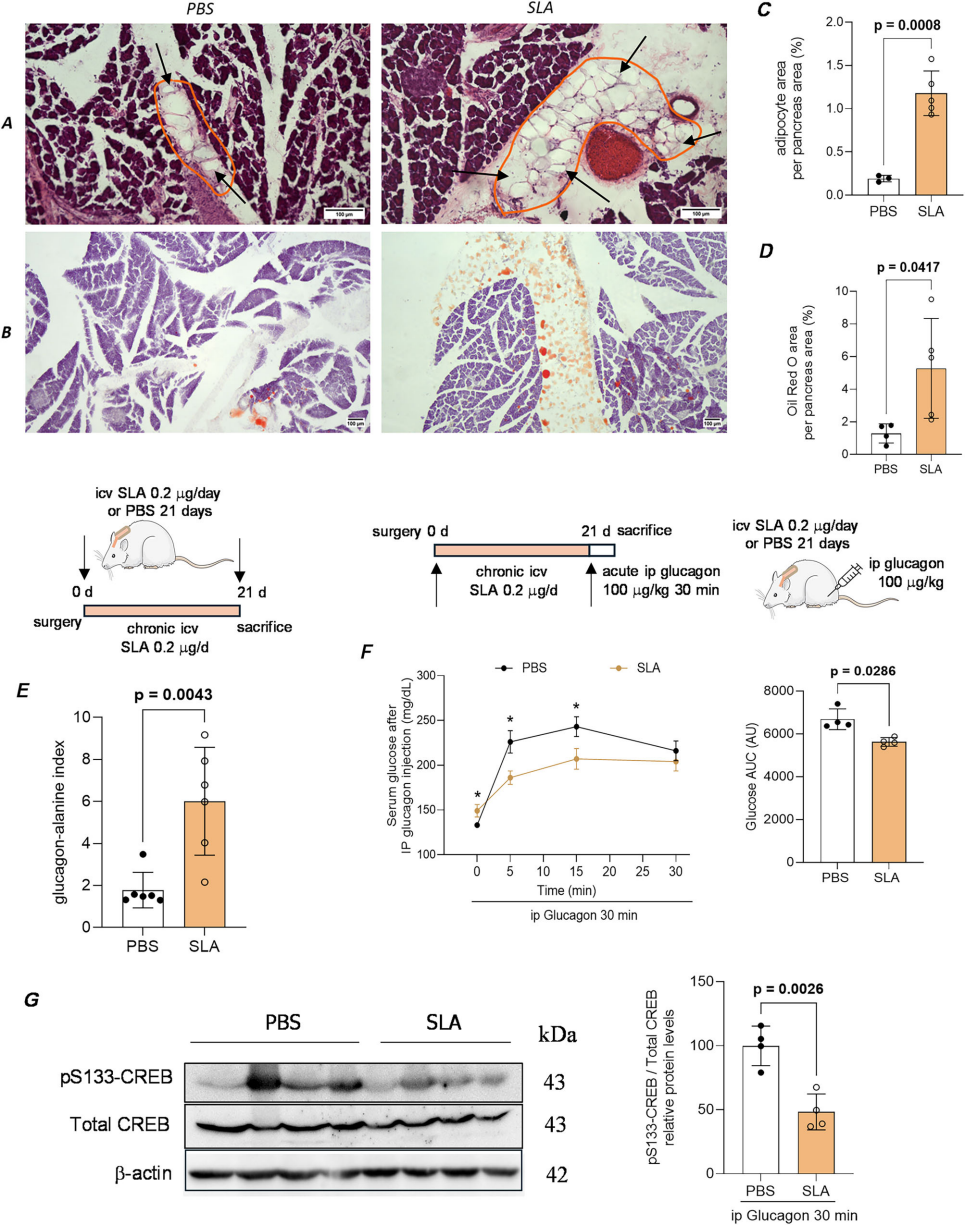
Central leptin signalling deficiency is associated with pancreatic adipocyte infiltration and hepatic glucagon resistance *A* and *B*, representative pancreatic sections stained with haematoxylin and eosin (*A*, 10×) and Oil Red O (*B*, 4×), showing interlobular adipocyte infiltration (arrows) and lipid accumulation. *C* and *D*, quantification of adipocyte area in haematoxylin and eosin‐stained sections (*C*) and lipid accumulation in Oil Red O–stained sections (*D*), expressed relative to total pancreatic tissue area. *E*, glucagon–alanine index in 16 h‐fasted rats after 21 days of PBS or SLA treatment. *F*, serum glucose levels during an i.p. glucagon challenge and corresponding area under the curve (AUC) in 16 h‐fasted rats. *G*, immunoblot and quantification of hepatic pSer133‐CREB and total CREB following glucagon administration. *n* = 4 (PBS) and *n* = 5 (SLA) (*C* and *D*); *n* = 6 per group (*E*); *n* = 4 per group (*F* and *G*). *F*, *P < 0.05. Data are presented as the mean ± SD. *P* values in bold indicate statistical significance.

**Figure A2:**
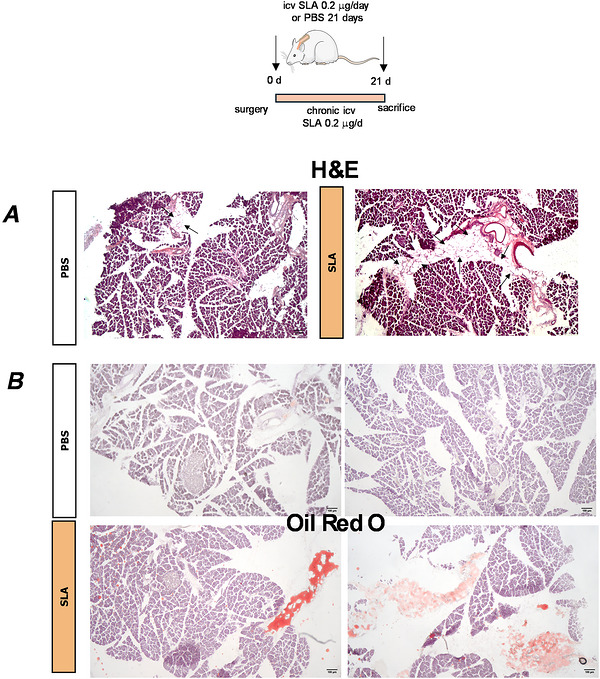
Hypothalamic leptin signalling deficiency promotes pancreatic adipocyte infiltration *A* and *B*, representative pancreatic sections stained with haematoxylin and eosin (*A*, 10× magnification) and Oil Red O staining (*B*, 4× magnification) showing intrapancreatic adipocyte infiltration (arrows) and lipid accumulation, both of which were markedly increased in SLA‐treated rats (*n* = 4 for PBS, *n* = 5 for SLA). Staining was performed in two sections per rat pancreas, spaced at least 200 µm apart.

Islet density and β‐cell mass did not differ between the PBS‐ and SLA‐infused groups (Fig. 5*A*–*C*). By contrast, SLA‐infused rats exhibited a significant increase in α‐cell mass and α‐cell area relative to islet area (Fig. 5*D*–*G*), consistent with elevated pancreatic glucagon content. Histological analysis revealed increased intrapancreatic adipocyte infiltration in SLA‐treated rats (Fig. 6*A* and *B*; see also Appendix, Fig. A2*A*, *B*), with both haematoxylin and eosin and Oil Red O staining demonstrating increased lipid deposition compared to controls (Fig. 6*C* and *D*). Fatty infiltration of the pancreas occurred concomitantly with α‐cell expansion, a phenotype previously associated with metabolic dysfunction and increased diabetes risk (Chan et al., 2022; Mahyoub et al., 2023).

### Reduced brain leptin signalling is associated with hepatic glucagon resistance

**Figure A3:**
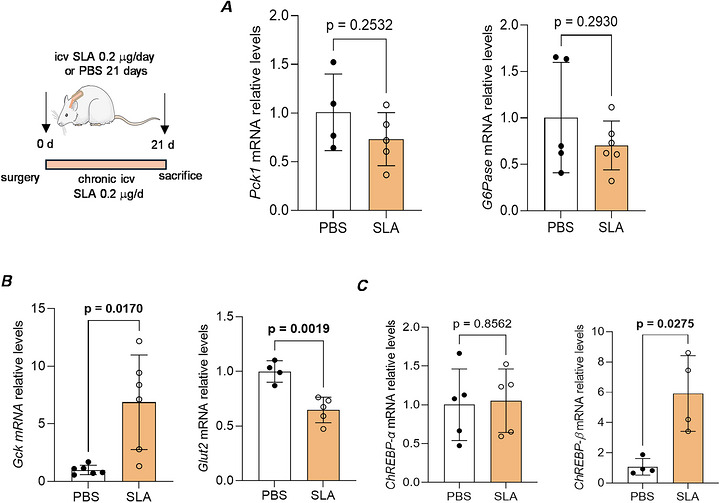
Deficient brain leptin signalling modifies hepatic gene expression regulating glucose metabolism *A*, hepatic mRNA expression of gluconeogenic genes (*Pck1* and *G6Pase*) after 21 days of central PBS or SLA infusion (*n* = 4–6 per group). *B*, hepatic mRNA expression of glycolytic genes (*Gck* and *Glut2*) (*n* = 4–6 per group). *C*, hepatic mRNA expression of transcription factors regulating glucose and lipid metabolism (*ChREBP‐α* and *ChREBP‐β*) (*n* = 4–5 per group). Data are the mean ± SD. *P* values in bold indicate statistical significance.

**Figure A4:**
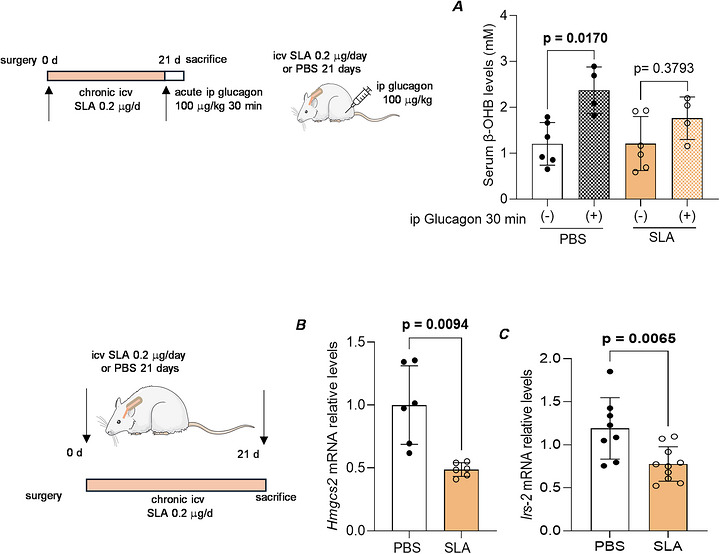
Central leptin signalling deficiency induces hepatic glucagon and insulin resistance and disrupts the gut‐liver‐brain axis *A*, β‐hydroxybutyrate (β‐OHB) levels after i.p. glucagon injection (100 µg kg^−1^ BW, 30 min) in fasting (16 h) rats after 21 days of central PBS or SLA (0.2 µg day^−1^) infusion (*n* = 6 per group in the absence or *n* = 4 per group in the presence of glucagon). *B*, hepatic mRNA expression levels of *Hmgcs2 (n* = 6 per group). *C*, hepatic mRNA expression levels of *Irs2* (*n* = 7 for PBS, *n = *10 for SLA). Data are the mean ± SD. *P* values in bold indicate statistical significance.

To assess hepatic glucagon responsiveness, the glucagon–alanine index, a marker of hepatic glucagon resistance (Wewer Albrechtsen et al., 2018), was measured. SLA‐infused rats exhibited an increased glucagon–alanine index (Fig. 6*E*). *In vivo* glucagon administration increased serum glucose levels and hepatic CREB phosphorylation in control rats, whereas both responses were markedly attenuated in SLA‐treated animals (Fig. 6*F* and *G*). Hepatic expression of gluconeogenic genes (*Pck1* and *G6pc*) tended to be reduced following SLA treatment (see Appendix, Fig. A3*A*). In addition, glucagon‐induced ketogenesis, assessed by circulating β‐hydroxybutyrate levels, was blunted in SLA‐infused rats (see Appendix, Fig. A4*A*), together with reduced expression of *Hmgcs2* (see Appendix, Fig. A4*B*). Hepatic expression of *Irs2* was also reduced (see Appendix, Fig. A4*C*), consistent with impaired hepatic insulin signalling.

### Hepatic lipid metabolism and inflammation

**Figure 7:**
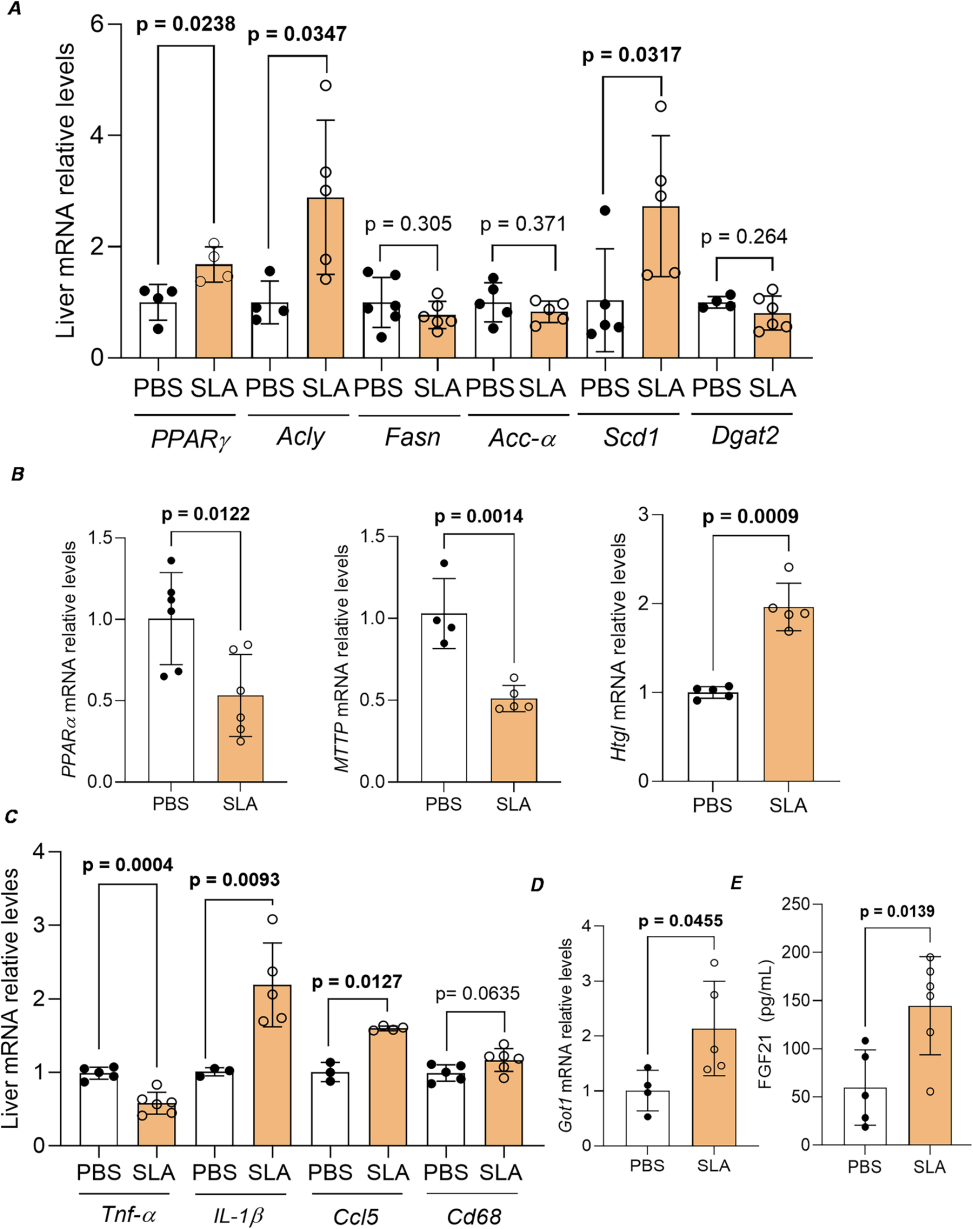
Central leptin signalling deficiency is associated with altered hepatic lipid metabolism–related gene expression and elevated circulating FGF21 *A*, hepatic mRNA expression of genes involved in de novo lipogenesis. *B*, hepatic mRNA expression of genes involved in fatty acid oxidation and triacylglycerol (TAG) metabolism. *C*, hepatic mRNA expression of proinflammatory cytokines. *D*, hepatic mRNA levels of *Got1*. *E*, Fasting serum FGF21 concentrations after 21 days of PBS or SLA treatment. *n* = 4–6 per group (*A*); *n* = 5–6 per group (*B*); *n* = 3–5 (PBS) and *n* = 4–6 (SLA) (*C*); *n* = 4–5 per group (*D*); *n* = 5–6 per group (*E*). Data are presented as the mean ± SD. *P* values in bold indicate statistical significance.

To evaluate hepatic lipid metabolism under conditions of attenuated central leptin signalling, expression of genes involved in lipid synthesis, oxidation and export was examined. SLA‐infused rats exhibited increased hepatic expression of *Acly*, *Pparγ* and *Scd1*, whereas *Fasn*, *Accα* and *Dgat2* expression was unchanged (Fig. 7*A*). By contrast, expression of *Pparα* and *Mttp* was markedly reduced, whereas *Htgl* expression was increased (Fig. 7*B*). Although the expression of *Tnfα* was reduced by SLA infusion, markers of hepatic inflammation and stress, including Il1β and Ccl5, were elevated (Fig. 7*C*). In parallel, Got1 expression was increased (Fig. 7*D*), consistent with early stress‐associated and profibrotic signalling rather than a classical tumour necrosis factor α‐driven inflammatory response (Li et al., 2017; Nov et al., 2010; Tanwar et al., 2020).

### Central SLA infusion is associated with increased circulating FGF21 levels

Serum FGF21 concentration was markedly elevated in SLA‐infused rats compared to PBS‐infused controls (Fig. 7*E*), supporting previous reports linking hepatic metabolic stress to increased FGF21 secretion (Stefan et al., 2023). In parallel, circulating levels of GIP and PYY were reduced in SLA‐infused animals (Table 1), comprising hormones known to interact with central metabolic circuits (Batterham et al., 2007; Müller et al., 2025).

### Decreased brain leptin signalling altered amino acid metabolism and reduced hepatic BCAA catabolism

**Figure 8:**
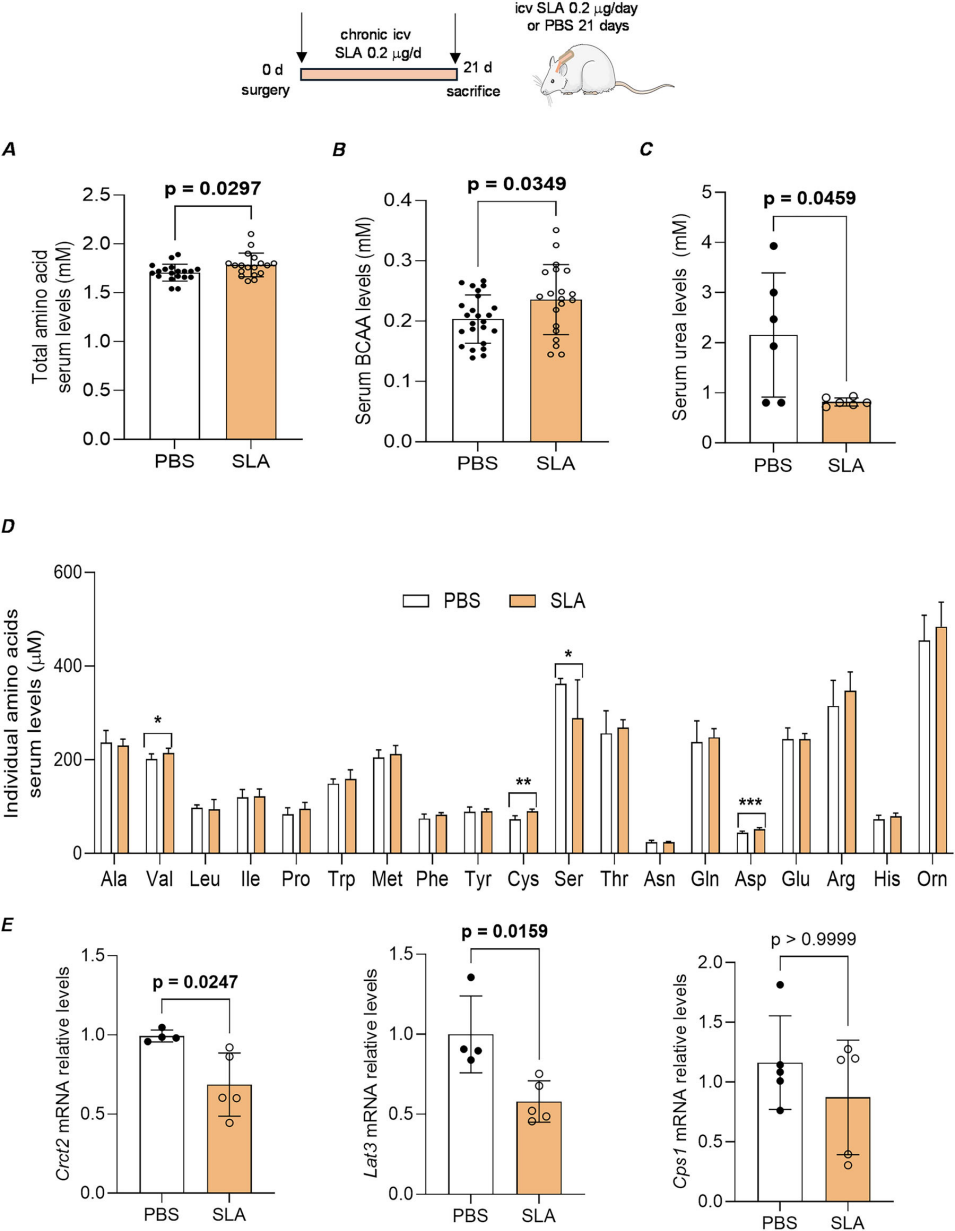
Central leptin signalling deficiency is associated with increased circulating amino acids and altered hepatic amino acid metabolism. *A*, total circulating amino acid concentrations. *B*, Circulating branched‐chain amino acid (BCAA) concentrations. *C*, serum urea levels. *D*, concentration of individual circulating amino acids. *E*, hepatic mRNA expression of genes involved in amino acid transport and catabolism. *n* = 18–19 per group (*A*); *n* = 18 per group (*B*); *n* = 6 per group (*C*); *n* = 6 (PBS) and *n* = 12 (SLA) (*D*); *n* = 4–5 (PBS) and *n* = 5 (SLA) (*E*). *D*, *P < 0.05, **P < 0.01, ***P < 0.001. Data are presented as the mean ± SD. *P* values in bold indicate statistical significance.

SLA‐infused rats exhibited elevated fasting serum concentrations of total and BCAAs, together with reduced serum urea levels (Fig. 8*A*–*C*), in line with impaired hepatic amino acid clearance (Galsgaard et al., 2018; Winther‐Sørensen et al., 2020). Several individual amino acids, including cysteine, aspartate and valine, were increased, whereas serine levels were reduced (Fig. 8*D*), a pattern associated with hepatic steatosis (Suppli et al., 2020; Wada et al., 2021). Hepatic expression of genes involved in amino acid transport and metabolism (*Crtc2*, *Slc43a1* and *Cps1*) was also reduced (Fig. 8*E*).

**Figure 9:**
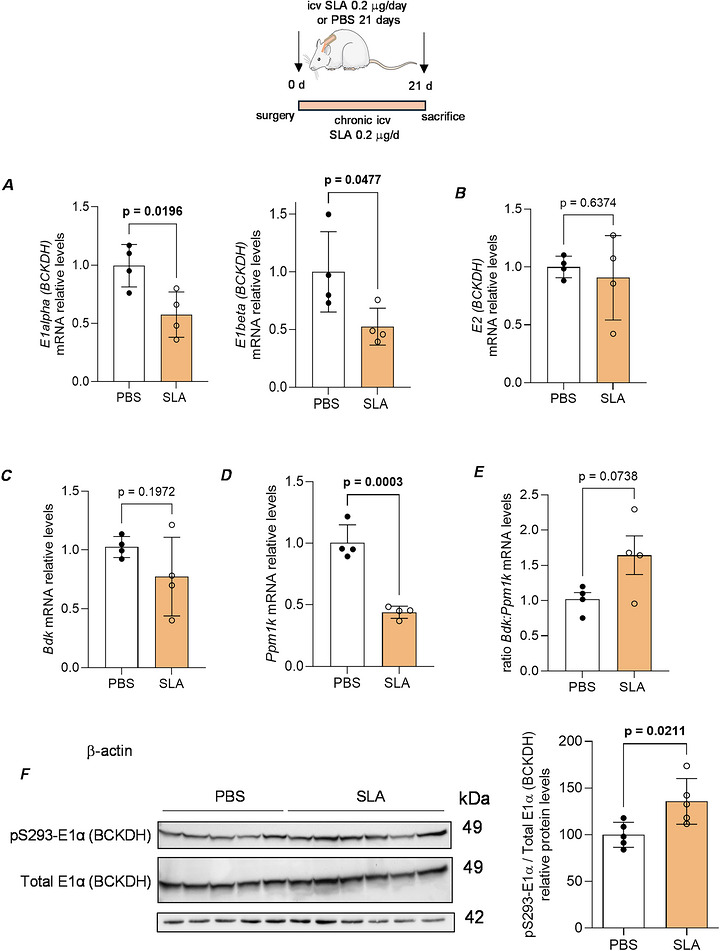
Brain leptin signalling deficiency is associated with reduced hepatic BCKDH activity and altered BCAA catabolism *A* and *B*, hepatic mRNA expression of the E1α and E1β (*A*) and E2 (*B*) subunits of the branched‐chain α‐ketoacid dehydrogenase (BCKDH) complex. *C*, hepatic mRNA levels of *Bdk* (BCKDH kinase). *D*, hepatic mRNA levels of *Ppm1k* (BCKDH phosphatase). *E*, hepatic *Bdk/Ppm1k* mRNA ratio. *F*, representative immunoblot and quantification of phosphorylated (pSer293) and total E1α BCKDH protein levels in liver. *n* = 4 per group (*A*–*E*); *n* = 5 (PBS) and *n* = 6 (SLA) (*F*). Data are presented as the mean ± SD. *P* values in bold indicate statistical significance.

Gene expression of components of the branched‐chain α‐ketoacid dehydrogenase (BCKDH) complex in the liver was altered following SLA administration. Brain SLA administration reduced the expression of *E1α* and *E1β*, decreased *Ppm1k*, and increased the *Bdk/Ppm1k* ratio (Fig. 9*A*–*E*), resulting in BCKDH inactivation, as evidenced by increased inhibitory phosphorylation of the E1α subunit at Ser293 (Fig. 9*F*), which supports the reduced hepatic BCAA catabolic capacity (Shin et al., 2014).

## Discussion

Animal models have been instrumental in elucidating the mechanisms underlying metabolic disease (Mashimo & Serikawa, 2009). Classic models such as *ob*/*ob*, *db*/*db* and *Irs2* knockout mice have provided fundamental insights into leptin and insulin signalling but represent fixed, monogenic defects associated with severe obesity (Kubota et al., 2000). Increasing evidence, however, indicates that impaired brain leptin signalling can disturb glucose and lipid homeostasis independently of adiposity, contributing to early metabolic dysfunction (Berglund et al., 2012). In this context, chronic attenuation of central leptin signalling in lean rats provides a physiologically relevant model to investigate systemic consequences of altered central metabolic control prior to overt obesity.

The data presented here demonstrate that, in lean, normoleptinemic rats, chronic SLA administration induced a constellation of alterations including modest increases in food intake and body weight, visceral adiposity, and lipid accumulation in liver and pancreas. These findings are consistent with the established anti‐steatotic actions of centrally mediated leptin signalling through autonomic pathways (Berglund et al., 2012; Bonzón‐Kulichenko et al., 2009; Gallardo et al., 2007; Hackl et al., 2019). Importantly, these changes cannot be explained solely by increased adiposity. Rather, they arise in the setting of disrupted central leptin action and precede the hallmark features of advanced adipose dysfunction. This temporal dissociation supports a model in which neuroendocrine imbalance acts as an initiating event, with adipose tissue serving as a secondary amplifier rather than the primary driver of metabolic deterioration.

A key novel finding is the coexistence of fasting and post‐load hyperglucagonemia with α‐cell expansion and impaired glucagon suppression. Elevated circulating amino acids, reduced hepatic BCKDH activity and downregulation of genes involved in amino acid transport and ureagenesis further indicate impaired hepatic amino acid clearance and emerging glucagon resistance. Together, these findings demonstrate dysregulation of the liver–α‐cell axis linking hepatic metabolic stress to increased glucagon output, a feature characteristic of early type 2 diabetes (Reaven et al., 1987; Wewer Albrechtsen et al., 2018; Winther‐Sørensen et al., 2020).

SLA‐infused rats also exhibited fasting hyperinsulinemia and impaired glucose tolerance despite preserved circulating leptin levels and only modest increases in caloric intake. These findings reinforce that reduced central leptin signalling perturbs insulin and glucagon regulation beyond the effects of caloric excess. Unlike β‐cell, specific leptin receptor knockout models (Covey et al., 2006), SLA‐infused rats displayed α‐cell expansion and pancreatic lipid accumulation, consistent with lipotoxic remodelling as proposed by Unger (Unger & Zhou, 2001). The coexistence of functional α‐cell insulin resistance, as described in αIRKO mice (Kawamori et al., 2009), together with hepatic glucagon resistance, further supports impaired inter‐organ co‐ordination and glucagon‐driven metabolic inflexibility. These observations are particularly relevant to non‐obese forms of type 2 diabetes, characterized by islet dysfunction and ectopic lipid deposition despite limited adiposity (Karaevren et al., 2025). Hence, the SLA model recapitulates several of these features, providing mechanistic insight into how early attenuation of central leptin signalling may precipitate endocrine remodelling and metabolic inflexibility resembling this phenotype.

**Figure 10:**
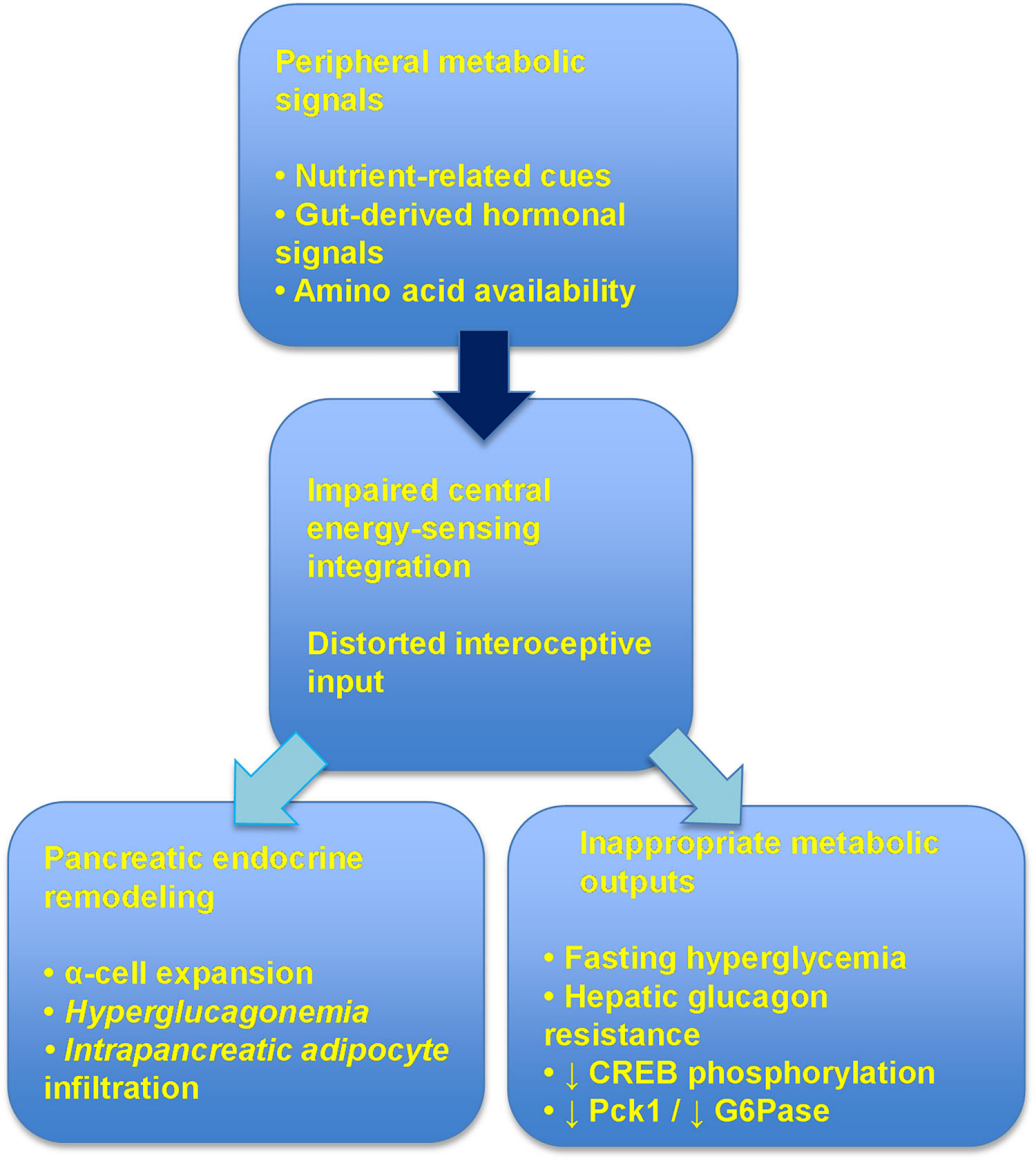
Hierarchical disruption model of gut–brain–pancreas–liver metabolic control in SLA‐treated rats The schematic illustrates a hierarchical model in which persistent alterations in peripheral metabolic signals lead to impaired central energy‐sensing integration. Distorted interoceptive input results in inappropriate autonomic and hormonal outputs affecting both pancreatic and hepatic function. Pancreatic endocrine remodelling, characterized by α‐cell expansion, hyperglucagonemia and intrapancreatic adipocyte infiltration, acts as an intermediate amplifier linking central dysfunction to hepatic glucagon resistance and fasting hyperglycaemia. These alterations occur despite reduced CREB phosphorylation and downregulation of canonical gluconeogenic genes (*Pck1* and *G6Pase*), providing a unifying framework for the dissociation between circulating glucose levels and hepatic gluconeogenic gene expression observed in SLA‐treated rats. Arrows indicate direction of information flow and hierarchical regulation rather than direct causal or endocrine pathways.

Emerging evidence underscores bidirectional liver–brain communication pathways that co‐ordinate metabolic responses. Vagal afferents in the hepatoportal region relay nutrient‐related signals to hypothalamic circuits, influencing glucose and lipid regulation (Berthoud et al., 2024; Hwang et al., 2025). Elevated circulating FGF21 levels in SLA‐infused rats are consistent with potential alterations in liver–brain communication and align with studies demonstrating that glucagon receptor deficiency induces hepatic lipid accumulation and increases FGF21 expression (Cacciottolo et al., 2025; Rose et al., 2025). Increased FGF21 levels have also been reported in metabolic syndrome and prediabetes (Tan et al., 2025). Concurrent reductions in GIP and PYY suggest altered enteroendocrine signalling, which may further contribute to systemic metabolic imbalance. These co‐ordinated alterations are integrated within the hierarchical conceptual framework illustrated in Fig. 10. Nevertheless, further studies are required to define causal mechanisms within this network.

This study has limitations. Some alterations may reflect secondary consequences of increased caloric intake rather than direct effects of impaired central leptin signalling. Although prior work demonstrates that central leptin action regulates glucose and lipid metabolism independently of adiposity (Bonzón‐Kulichenko et al., 2009, 2011; Hackl et al., 2019), inclusion of a pair‐fed SLA group would strengthen causal inference. However, the Lee index did not differ between groups, suggesting comparable adiposity. An additional limitation relates to the sample size in some gene expression analyses, where a low number of animals per group may reduce statistical power. Although these analyses were conducted in independent cohorts and yielded consistent directional changes across multiple end‐points, the relatively small sample size in these assays should be considered when interpreting the findings. Future studies with larger sample sizes will be important to confirm these observations. Finally, although the results support altered glucagon regulation, direct interrogation of autonomic pathways will be necessary to delineate neural contributions.

In conclusion, chronic attenuation of brain leptin signalling in lean rats induces co‐ordinated endocrine and metabolic disturbances, including hyperglucagonemia, hepatic glucagon resistance, impaired glucose tolerance and reduced hepatic BCAA catabolism, despite preserved circulating leptin levels. These findings support the concept that central neuroendocrine dysregulation can precede overt obesity and drive systemic metabolic inflexibility. The SLA model therefore provides a tractable experimental framework to investigate early neuroendocrine mechanisms linking impaired brain leptin action to metabolic dysfunction relevant to non‐obese type 2 diabetes (Tobias et al., 2014).

## Additional information

## Competing interests

The authors declare that they have no competing interests.

## Author contributions

A.A., E.B.‐M. and N.G. were responsible for conceptualization. C.P., L.M., B.M., E.C.‐A., G.P., I.C.‐C., I.R. and J.P. were responsible for methodology. C.P., L.M., Ó.G., B.R., S.A.‐J., E.M., C.A., B.M., E.C.‐A., G.P., I.C.‐C., I.R., J.P., A.A. and N.G. were responsible for investigations and formal analysis. A.A. and N.G. were responsible for writing the original draft. C.P., L.M., Ó.G., E.M., E.B.‐R., C.A., G.P., I.C.‐C., E.B.‐M., A.A. and N.G. were responsible for reviewing and editing. J.P., C.A., G.P., I.C.C., A.A. and N.G. were responsible for supervision. A.A. and N.G. were responsible for funding acquisition.

## Funding

This article was funded by Ministerio de Ciencia e Innovación (MCIN): ANTONIO ANDRES, RTI2018‐098643‐B‐I00; Ministerio de Ciencia e Innovación (MCIN): NILDA, GALLARDO, PID2021‐128243OB‐I00; Universidad de Castilla‐La Mancha (UCLM): ANTONIO ANDRES, 2021 GRIN‐30987; Universidad de Castilla‐La Mancha (UCLM): ANTONIO ANDRES, 2022‐GRIN‐34280; Ministerio de Ciencia e Innovación (MCIN): SARA ARTIGAS‐JERÓNIMO, FJC2021‐047764‐I; U.S. Department of Veterans Affairs (VA): ERNESTO BERNALMIZRACHI, BX002728; HHS | National Institutes of Health (NIH): ERNESTO BERNAL‐MIZRACHI, R01‐DK073716; HHS | National Institutes of Health (NIH): ERNESTO BERNAL‐MIZRACHI, DK132103; and HHS | National Institutes of Health (NIH): ERNESTO BERNAL‐MIZRACHI, DK133183.

## Supporting information




Statistical Summary Document

Peer Review History

Supporting Information

## Data Availability

The data supporting the findings of this study are available within the article and its supporting material. Additional data are available from the corresponding author upon reasonable request.

## Appendix A

**Table A1:** TaqMan probes for real‐time quantitative PCR

Gene	ABI assay ID
*Acc‐α*	Rn00573474_m1
*Acly*	Rn00566911_m1
*Ccl5*	Rn00579590_m1
*Cd68*	Rn014956334_g1
*Dgat‐2*	Rn01506781_m1
*Fasn*	Rn01463556_g1
*G6Pase*	Rn00598561_m1
*Gk*	Rn01443433_m1
*Glut2*	Rn00563565_m1
*Hmgcs2*	Rn00597339_m1
*Htgl*	Rn01530834_m1
*Il‐1b*	Rn01514151_m1
*Irs‐2*	Rn001478821_s1
*Mttp*	Rn01522974_m1
*Pck1*	Rn01529009_g1
*Pomc*	Rn00595020_m1
*Pparα*	Rn00566193_m1
*Pparγ*	Rn00440945_m1
*Scd1*	Rn00594894_g1
*Socs3*	Rn00585674_s1
*Tnf‐α*	Rn99999017_m1
*18Sr RNA*	4319413E

**Table A2:** Primer sequences for real‐time quantitative PCR.

Gene	Forward sequence (5′‐ to 3′)	Reverse sequence (5′‐ to 3′)
*Agrp*	TCTTCCACCTTTGCAGCATT	TGTAGCCAGGGCATGAGG
*Bdk*	GTCATCACCATCGCCMTMCG	TGTGGTGMGTGGTAGTCCATG
*ChREBP‐α*	AGGCTCAAGCATTCGAAGAG	TGCATCGATCACAGGTCATT
*ChREBP‐β*	CTTGTCCCGGCATAGCAAC	TCTGCAGATCGCGCGGAG
*Cps1*	TCTGGGCATGGGTGGCCAGA	TGGGACAGATGCCGGAGCCT
*Crtc2*	GAGTACCTGGCTTTGAGGTGTCA	CATGCGCAACTCATCTTCCA
*E1alpha*	TGCTGCCACCTTGGAGTGTCCT	GCCGTCGGGCCTCCTTAGTG
*E1beta*	CGCCTTTGACAGGCGGCTTT	ATTCGCAGCTCGTCAGCCAGT
*E2*	CCGGAACGCTGTCAGATTGACCT	ACG CCA TCG TAA GGG CTG GT
*Got1*	GAAGACAATGGCTGACCGGA	AGGTTCTTGGTGGTCAAGCC
*Lat3*	CTGGGGATTCTCATGGACCG	GCCAGCAAACCCATTCAAGG
*Ppm1k*	TTTGGGTTCGCACAGTTGAC	AAGTCTTTCTCCCGAGGAAGC
*18S rRNA*	CGGCTACCACATCCAAGGAA	GCTGGAATTACCGCGGCT
