# Supplementary material for: Chronic attenuation of brain leptin signalling is associated with early metabolic dysfunction in lean rats
Source: J Physiol. 2026 May 7;604(12):4717–44. doi: 10.1113/JP290832 (PMC13267677; doi:10.1113/JP290832)
Supplement: Supplementary file 3 — Supporting Information [file TJP-604-4717-s003.pdf]

Supporting information

Uncropped images corresponding to Figure 2*A*

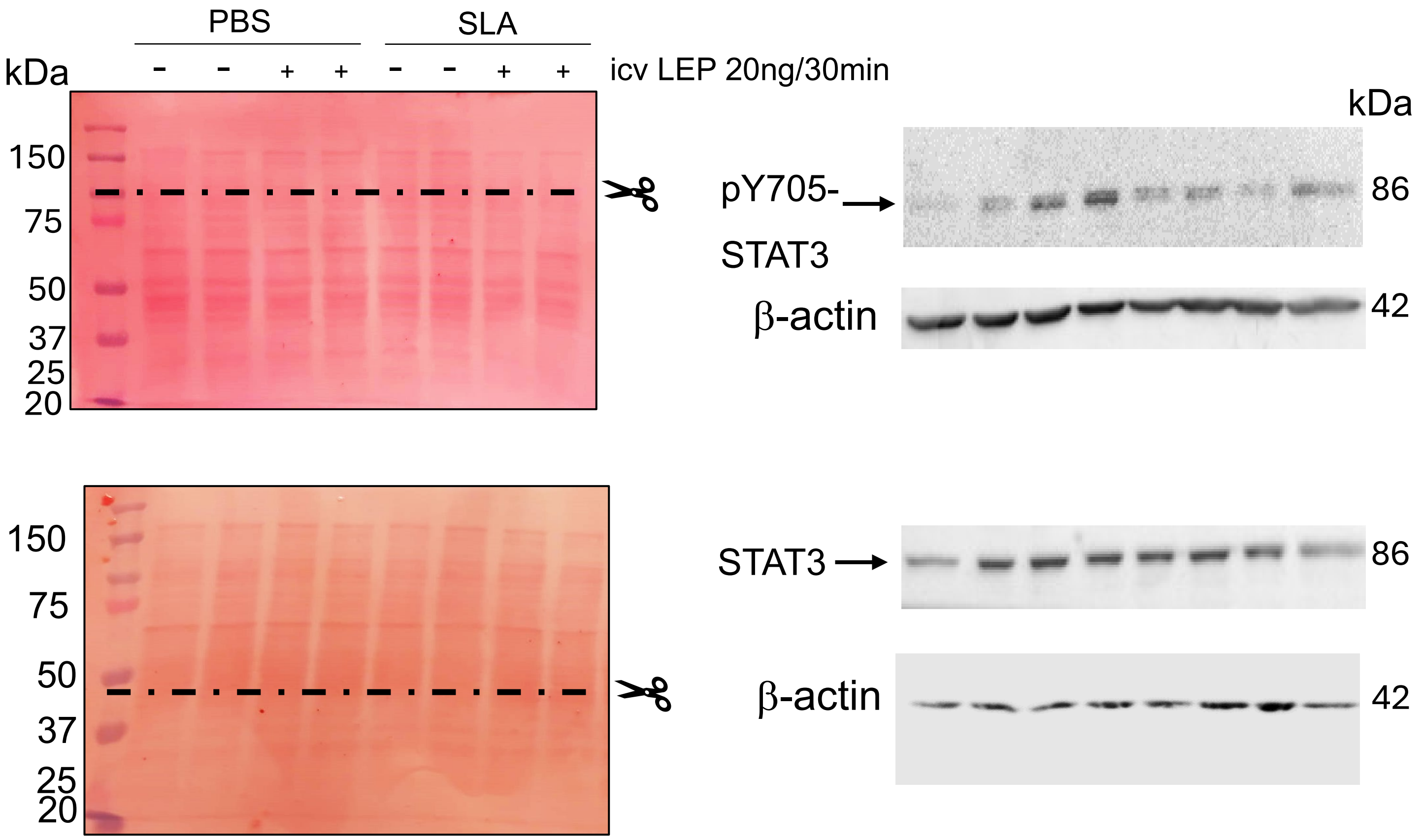

Supplementary Figure 1 - Ponceau red staining and full Western Blot membranes of proteins obtained from control (PBS) and SLA-infused rats for 21 days in support of Figure 2*A*. Data shown the phosphorylation of STAT3 (pY-705) and total STAT3 protein levels in the hypothalamus in response to icv leptin infusion (20 ng/30 min) or vehicle in fasted (16h) rats treated icv with SLA or PBS for 21 days (n = 4 rats per group). Uncropped images corresponding to Figure 2*A*. Membranes were cut around 75 kDa before probing with antibodies to allow concomitant detection of more than one protein on the same membrane.

Uncropped images corresponding to Figure 6*G*

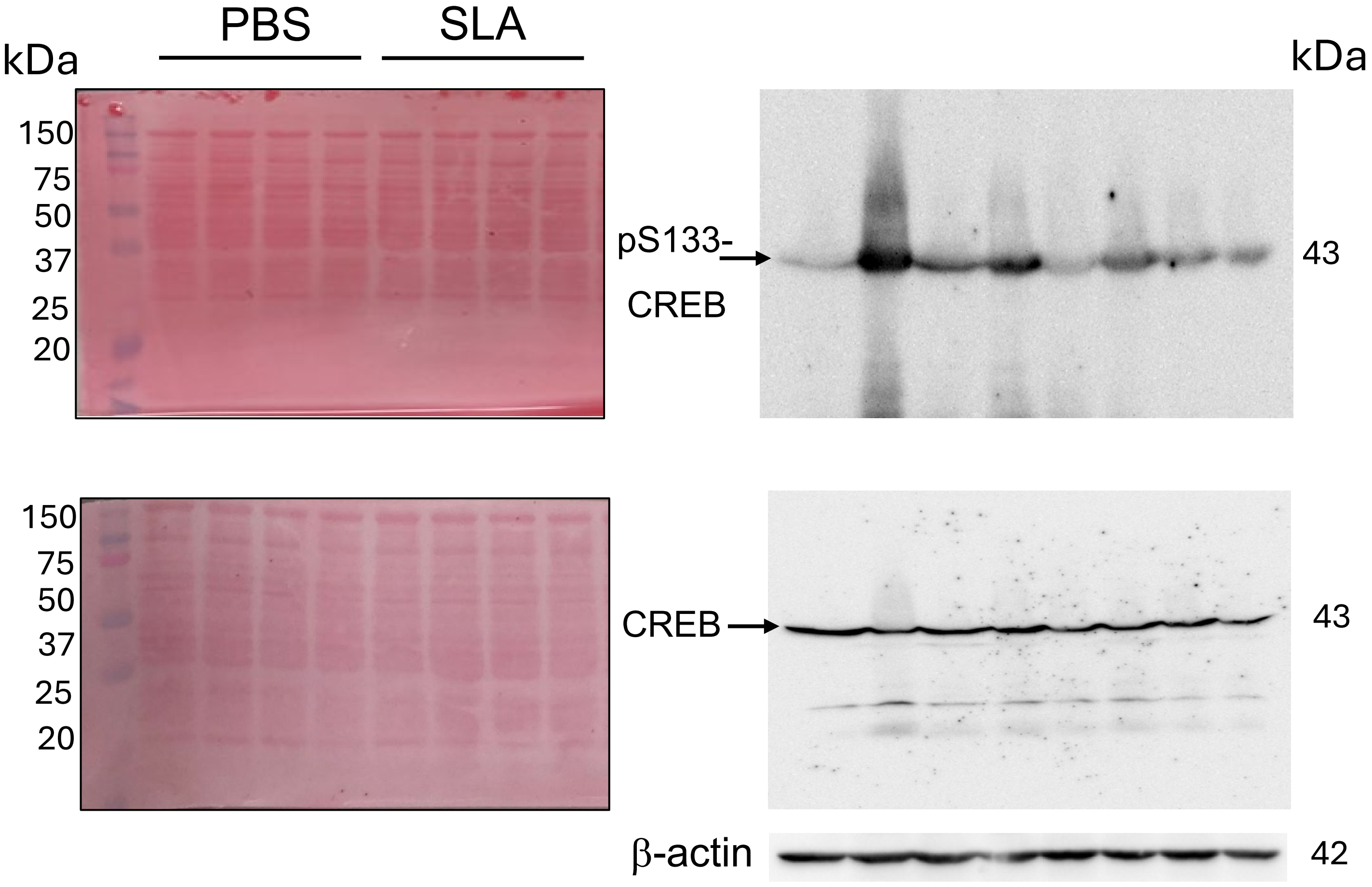

Supplementary Figure 2 - Ponceau red staining and full Western Blot membranes of proteins obtained from control (PBS) and SLA-infused rats for 21 days in support of Figure 6*G*. Data shown the phosphorylation of CREB (pS-133) and total CREB protein levels in the liver after intraperitoneal glucagon injection (100 μg/kg BW) for 30 min in fasted (16h) rats treated icv with SLA or PBS for 21 days (n = 4 rats per group). Uncropped images corresponding to Figure 6*G*.

Uncropped images corresponding to Figure 9*F*

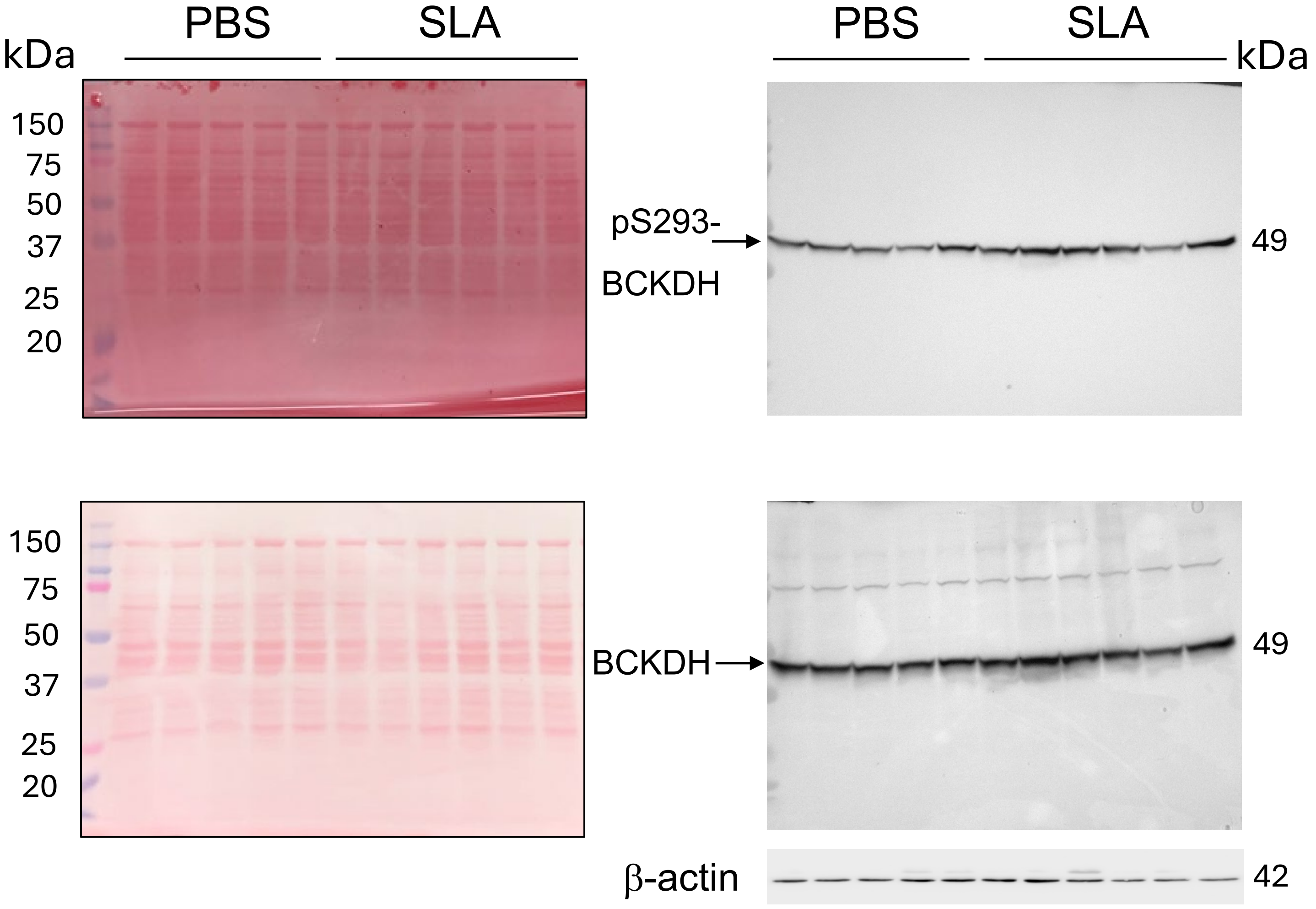

Supplementary Figure 3 - Ponceau red staining and full Western Blot membranes of proteins obtained from control (PBS) and SLA-infused rats for 21 days in support of Figure 9*F*. Data shown the phosphorylation of BCKDH-E1α subunit (pS-133) and total BCKDH-E1α protein levels in the liver as an index of BCKDH inactivation in fasted (16) rats treated icv with SLA or PBS for 21 days (n = 5 per PBS; n = 6 per SLA). Uncropped images corresponding to Figure 9*F*.
