# Supplementary material for: Chronic attenuation of brain leptin signalling is associated with early metabolic dysfunction in lean rats
Source: J Physiol. 2026 May 7;604(12):4717–44. doi: 10.1113/JP290832 (PMC13267677; doi:10.1113/JP290832)
Supplement: Supplementary file 2 — Peer Review History [file TJP-604-4717-s002.pdf]

The Journal of Physiology

Chronic Attenuation of Brain Leptin Signaling Is Associated with Early Metabolic Dysfunction in Lean Rats

CRISTINA PINTADO, LORENA MAZUECOS, ÓSCAR GÓMEZ-TORRES, BEATRIZ MERINO, ELENA CASANUEVA-ÁLVAREZ, BLANCA RUBIO, EMMA BURGOS-RAMOS, SARA ARTIGAS-JERÓNIMO, EDUARDO MOLTÓ, INÉS MARÍA RAMOS, JUSTA MARÍA POVEDA, GERMÁN PERDOMO, IRENE CÓZAR-CASTELLANO, CARMEN ARRIBAS, ERNESTO BERNAL-MIZRACHI, ANTONIO ANDRES, and NILDA GALLARDO
DOI: 10.1113/JP290832

Corresponding author(s): ANTONIO ANDRES (antonio.andres@uclm.es)

The following individual(s) involved in review of this submission have agreed to reveal their identity: Vsevolod Y Polotsky (Referee #2)

Review Timeline:

Submission Date:	14-Jan-2026
Editorial Decision:	10-Feb-2026
Revision Received:	09-Mar-2026
Editorial Decision:	23-Mar-2026
Revision Received:	25-Mar-2026
Accepted:	13-Apr-2026

Senior Editor: Bettina Mittendorfer

Reviewing Editor: Bettina Mittendorfer

Transaction Report:

(Note: With the exception of the correction of typographical or spelling errors that could be a source of ambiguity, letters and reports are not edited. Depending on transfer agreements, referee reports obtained elsewhere may or may not be included in this compilation. Referee reports are anonymous unless the Referee chooses to sign their reports.)

Dear Dr ANDRES,

Re: JP-RP-2026-290832 "**Chronic Attenuation of Brain Leptin Signaling Is Associated with Early Metabolic Dysfunction in Lean Rats**" by CRISTINA PINTADO, LORENA MAZUECOS, ÓSCAR GÓMEZ-TORRES, BEATRIZ MERINO, ELENA CASANUEVA-ÁLVAREZ, BLANCA RUBIO, EMMA BURGOS-RAMOS, SARA ARTIGAS-JERÓNIMO, EDUARDO MOLTÓ, INÉS MARÍA RAMOS, JUSTA MARÍA POVEDA, GERMÁN PERDOMO, IRENE CÓZAR-CASTELLANO, CARMEN ARRIBAS, ERNESTO BERNAL-MIZRACHI, ANTONIO ANDRES, and NILDA GALLARDO

Thank you for submitting your manuscript to The Journal of Physiology. It has been assessed by a Reviewing Editor and by 2 expert referees and we are pleased to tell you that it is acceptable for publication following satisfactory revision.

The referee reports are copied at the end of this email.

Please address all the points raised and incorporate all requested revisions or explain in your Response to Referees why a change has not been made. We hope you will find the comments helpful and that you will be able to return your revised manuscript within 4 weeks. If you require longer than this, please contact journal staff: jp@physoc.org.

Your revised manuscript should be submitted online using the link in your Author Tasks Link Not Available. This link is accessible via your account as Corresponding Author; it is not available to your co-authors. If this presents a problem, please contact journal staff (jp@physoc.org). Image files from the previous version are retained on the system. Please ensure you replace or remove any files that are being revised.

If you do not wish to submit a revised version of your manuscript, you must inform our journal staff (jp@physoc.org) or reply to this email to request withdrawal. Please note that a manuscript must be formally withdrawn from the peer review process at one journal before it may be submitted to another journal.

TRANSPARENT PEER REVIEW POLICY: To improve the transparency of its peer review process The Journal of Physiology publishes online, as supporting information, the peer review history of all articles accepted for publication. Readers will have access to decision letters, including Editors' comments and referee reports, for each version of the manuscript, as well as any author responses to peer review comments. Referees can decide whether or not they wish to be named on the peer review history document.

ABSTRACT FIGURES: Authors are expected to use The Journal's premium BioRender account to create/redraw their Abstract Figures. Information on how to access this account is here: https://physoc.onlinelibrary.wiley.com/journal/14697793/biorender-access.

This will enable Authors to create and download high-resolution figures. If authors have used the free BioRender service, they can use the instructions provided in the link above to download a high-resolution version suitable for publication.

The link provided should only be used for the purposes of this submission. Authors will be charged for figures created on this account if they are not related to this manuscript submission.

LANGUAGE EDITING AND SUPPORT FOR PUBLICATION: If you would like help with English language editing, or other article preparation support, Wiley Editing Services offers expert help, including English Language Editing, as well as translation, manuscript formatting, and figure formatting at www.wileyauthors.com/eeo/preparation. You can also find resources for Preparing Your Article for general guidance about writing and preparing your manuscript at www.wileyauthors.com/eeo/prepresources.

RIGOUR AND REPRODUCIBILITY: We recommend authors consult The Journal's Rigour and Reproducibility requirements to ensure that their manuscript is suitable for publication: https://physoc.onlinelibrary.wiley.com/pb-assets/hub-assets/physoc/documents/TJP-Rigour-and-Reproducibility-Requirements.pdf

REVISION CHECKLIST:

Check that your Methods section conforms to journal policy: https://jp.msubmit.net/cgi-bin/main.plex?form_type=display_requirements#methods

Check that data presented conforms to the statistics policy: https://jp.msubmit.net/cgi-bin/main.plex?form_type=display_requirements#statistics

Upload a full Response to Referees file. To create your 'Response to Referees' copy all the reports, including any comments from the Senior and Reviewing Editors, into a Microsoft Word, or similar, file and respond to each point, using font or background colour to distinguish comments and responses and upload as the required file type.

Please upload two versions of your manuscript text: one with all relevant changes highlighted and one clean version with no changes tracked. The manuscript file should include all tables and figure legends, but each figure/graph should be uploaded

as separate, high-resolution files. The journal is now integrated with Wiley's Image Checking service. For further details, see: https://www.wiley.com/en-us/network/publishing/research-publishing/trending-stories/upholding-image-integrity-wileys-image-screening-service

You may also upload:

- 'Potential Cover Art' for consideration as the issue's cover image
- Appropriate Supporting Information (Video, audio or data set: see https://jp.msubmit.net/cgi-bin/main.plex?form_type=display_requirements#supp).

We look forward to receiving your revised submission.

If you have any queries, please reply to this email and we will be pleased to advise.

Yours sincerely,

Bettina Mittendorfer
Senior Editor
The Journal of Physiology

REQUIRED ITEMS

EDITOR COMMENTS

Reviewing Editor:

Senior Editor:

Comments to the Author:
Both reviewers felt this paper makes an important contribution but raised some issues that require attention.

REFEREE COMMENTS

Referee #1:

GENERAL COMMENTS

This was an interesting and generally well-written manuscript that describes a set of experiments that demonstrate the physiological impact of impaired leptin signalling. My main issues with the manuscript relate to the methods, in particular the fact that "no power calculations were performed", which makes it difficult to determine if the study was sufficiently powered (and therefore judge the validity of the conclusions). The numbers of animals per group, how groups were determined and numbers of animals included in each individual analysis should be described in the methods, not just in Figure legends. A diagram which shows this would be helpful.

SPECIFIC COMMENTS

METHODS

1. Would be good to include a brief explanation of Study 1 (even if it has been recently described)

2. Numbers of animals and details of number of animals per group, any premature losses of animals etc. needs to be described in the text.

3. Line 158 - can the authors provide further clarity re 'different groups of overnight fasted rats'? What is meant by 'different

groups'? How were these groups determined and how long was the fast?

4. Line 159 - How was this dose determined? If this was informed by a previous study, please provide a reference

5. Line 163 - again, justification of dose required

6. Line 164 - a brief description of the method (In addition to reference to previous study) would be helpful

7. Line 166 - Were the animals anesthetized or euthanised by CO2 injection (with death confirmed by decapitation)?

8. Line 219 (Gene expression analysis) - can the authors confirm that the selection of housekeeper genes followed the approved methodology? For the SYBR-green analyses - note that only 1 housekeeper was used, which is contrary to recommendations, so additional justification for this should be provided.

9. Statistical Analyses - why were no a priori power calculations carried out (this is typically a requirement for animal ethics applications/approvals)? Have the authors confirmed post-hoc that they had sufficient statistical power to support their conclusions?

RESULTS

10. The results section includes quite a bit of explanation about rationale for certain experiments and/or interpretation of the result, some of which is somewhat repetitive - suggest reducing/deleting (e.g. Line 244 - 247, Line 284 - 285, Line 320 - 322).

11. Sufficient to show significance to 3 sig figures

12. Line 346 - typo 'an increased Bdk/Ppm1k ratio' should be 'and increased....'?

13. Figures need to be labelled at the top of the page.

14. There are a lot of Figures/graphs - would consider showing some of this information in a table form instead, and reserve graphs/figures for the key findings (for added emphasis)

DISCUSSION

15. The discussion is generally well-written, however the organisation of the paragraphs makes is confusing at times. Suggest reviewing this, and ensuring that the interpretation of the relevant findings and the implications/relevance of these are included in the same paragraph.

16. Limitations of the study should also include reference to the animal numbers and statistical power (note earlier comments). Some of the gene expression graphs seem to suggest that there are only 3-4 animals per group (e.g. PPARalpha and Hgl mRNA in Figure 6. Is this really sufficient to draw robust conclusions?

Referee #2:

The paper addressed an important physiologic question if sustained attenuation of leptin receptor signaling in the brain in lean rats induces metabolic dysfunction. Adult rats received chronic IVC infusions of a rat-specific leptin receptor antagonist SLA vs vehicle for 21 day. The infusions consistently suppressed pSTAT3 signaling in the hypothalamus increasing Agrp and decreasing Socs3 and POMC gene expression, increased visceral adiposity, food intake, induced hepatic steatosis and pancreatic lipid accumulation, hyperinsulinemia and pancreatic lipid accumulation and hyperglucagonemia. Interestingly, hyperglucagonemia did not induce gluconeogenesis enzymes. In contrary, gluconeogenesis genes and CREB phosphorylation decreased, which suggest hepatic glucagon resistance. There was also evidence of disrupted glucagon-amino acid signaling manifested by elevated serum total and branched-chain amino acid levels. All these changes occurred without any changes in serum leptin and minimal increase in body weight without changes in the Obesity Lee index (Rat BMI). The authors conclude that attenuation of brain leptin signaling leads to metabolic dysfunction prior to obesity development.

The paper represents an important advance in our knowledge and it is scientifically sound.

Nevertheless, there are several important issues need to be addressed to increase these findings impact:

Major critique: 1) It would be important to assess energy expenditure and overall activity in view of the profound metabolic shifts. VO2/VCO2 and RER measurements would strengthen the manuscript. Did the core body temperature change?

2) Did the muscle mass decrease after SLA treatment? Were there any changes in subcutaneous adiposity and the amount of brown fat?

3) There was a slight increase in body weight without any change in the Obesity Lee Index: was there any change in body length?

4) How do the authors explain fasting hyperglycemia after SLA treatment despite suppressed gluconeogenesis?

5) Statistical Analysis - did you check your data for normality? If the data are not normally distributed, non-parametric statistics should be used

Minor critique:

1) Can you describe how you estimated liver glucagon signaling rather than providing a reference? - Line 164

2) In the results - line 250, include a reference to Fig. 1A

3) Line 264-265 - change to 'no significant change in glycogen and corticosterone levels'

4) Line 280: the C-peptide/insulin ratio reflects hepatic insulin clearance not IR. Please re-word

5) Line 325 - Add that TNF-a expression is decreased. It suggests that the data on a pro-inflammatory effect are equivocal

---

END OF COMMENTS

RESPONSE TO REFEREES

EDITOR COMMENTS

Reviewing Editor:

Senior Editor:

Comments to the Author:
Both reviewers felt this paper makes an important contribution but raised some issues that require attention.

We highly appreciate the Editors and Referees for their kind comments and for providing insightful, constructive suggestions, all of which are valuable and helpful for revising and improving the manuscript. We would also like to thank you for the effort to identify several aspects of the work that require modifications. Accordingly, the manuscript has been revised following the suggestions of the Referees. All changes included in the revised version of the manuscript are highlighted in red font.

REFEREE COMMENTS

Referee #1

GENERAL COMMENTS

This was an interesting and generally well-written manuscript that describes a set of experiments that demonstrate the physiological impact of impaired leptin signalling. My main issues with the manuscript relate to the methods, in particular the fact that "no power calculations were performed", which makes it difficult to determine if the study was sufficiently powered (and therefore judge the validity of the conclusions). The numbers of animals per group, how groups were determined and numbers of animals included in each individual analysis should be described in the methods, not just in Figure legends. A diagram which shows this would be helpful.

We greatly appreciate your careful review of our manuscript, as well as your encouraging comments and suggestions, which have been invaluable in helping us revise and improve the manuscript. We also thank the Referee for emphasizing the importance of transparency regarding animal numbers, sample size determination and statistical considerations.

In this sense, we understand the Referee’s concern regarding whether the study was sufficiently powered. In the Statistical Analysis section, we mistakenly stated that no *a priori* power calculations were performed to predetermine the sample sizes, when we intended to emphasize that the sample sizes were consistent with those used in our previous studies and in studies conducted by other groups. In fact, as noted by the Referee (question 9 – Statistical Analysis), an a priori sample size calculation was performed prior to the initiation of the study, as required for approval by both the Institutional Animal Ethics Committee and the regional governmental authority. The calculation was conducted using the GRANMO Sample Size and Power Calculator (Version 7.12, Municipal Institute of Medical Research of Barcelona), accepting an

alpha risk of 0.05 and a beta risk of 0.2 (power = 80%) in a two-sided test (information now included at the end of Ethical approval section in the revised version of the manuscript). Therefore, to prevent misinterpretation, this sentence has been modified in the revised manuscript (see further details in our response to your comment 9: Statistical Analysis). Besides, to accomplish the goals of the statistical policy, all data are now expressed as mean ± SD. Finally, following the Referee´s suggestion, we have included a new figure in the Methods (Experimental model section) illustrating an experimental overview of animals included in the study (Figure 1).

SPECIFIC COMMENTS

METHODS

1. Would be good to include a brief explanation of Study 1 (even if it has been recently described)

R1. We thank the Referee for this comment. As suggested, we have now incorporated the extended Methods information into the main manuscript. This material was previously included as Supplemental Material in our earlier version of the manuscript and has now been amended to include the number of animals per group, as follows:

Study 1: Chronic intracerebroventricular (icv) infusion of leptin receptor antagonist (SLA)

Intracerebroventricular infusion of leptin receptor antagonist was performed as described previously (Mazuecos et al., 2024). Rats were anesthetized by inhalation of a mixture of O_2 and isoflurane (Pharmacia-Upjohn, Barcelona, Spain) and placed in a stereotaxic frame (David Kopf, Tujunga, CA, USA) with a thermal blanket underneath. An opening in the skull was made with a dental drill at – 1.6 mm lateral to the midline and 0.8 mm anterior to the bregma. Then a cannula connected to an osmotic minipump (Alzet, Palo Alto, CA) was implanted in the left ventricle for the chronic infusion for 21 days of SLA (0.2 µg/day) (rat-specific superactive leptin receptor antagonist, D23L/L39A/D40A/F41A, MBS400108, MyBiosource, San Diego, CA, USA), or its vehicle (PBS). Osmotic minipumps, with a release rate of 0.25 µl/h, were filled with 0.0332 µg/µl rat SLA or PBS. After all surgeries, rats were randomly housed individually for daily control of food intake (FI) and body weight (BW), thereby reducing variability in weight, serum levels of hormones, and metabolites depending on the amount of food eaten. The total number of rats used in this study was 48 (n = 24 rats per group treated with PBS or SLA) and euthanised after 21 days of treatment.

2. Numbers of animals and details of number of animals per group, any premature losses of animals etc. needs to be described in the text.

R2. As suggested, this information has been included in the revised version of the manuscript. (p. x - line x, p. y – line y,…)

3. Line 158 - can the authors provide further clarity re 'different groups of overnight fasted rats'? What is meant by 'different groups'? How were these groups determined and how long was the fast?

R3.1. We thank the Referee for pointing out the lack of clarity in this sentence. As commented before (A1), we have now incorporated the extended Methods information into the main manuscript. As a result, this sentence has been amended as follows:

Study 2: ICV treatment with SLA or PBS for 21 days followed by an acute central leptin infusion

Additionally, on a subset of each group (n = 8 per group) we performed an acute brain leptin signaling stimulation at the end of the 21-day infusion with PBS or SLA. To this end, a guide cannula was pre-implanted, at day 0, in the contralateral ventricle for the posterior acute infusion of leptin or PBS. A dummy cannula was inserted into the guide cannula and secured in place by screwing it onto the guide to prevent contamination. After 21 days of chronic icv SLA or PBS infusion, overnight fasted (16h) rats were transiently anesthetized by inhalation of a mixture of O_2 and isoflurane and acutely treated with 2 µl of either rat leptin (20 ng) (LEP) (Sigma-Aldrich, St. Louis, MO, USA) or its vehicle (PBS). The animals were allowed to recover, and 30 minutes later, rats were euthanised by CO_2 inhalation and sacrificed by decapitation. Blood was removed and centrifuged (2000 xg, 15 min), and serum was recovered and frozen in liquid nitrogen for storage at –80ºC until use. The whole hypothalamus, liver, and epididymal and retroperitoneal fat pads were carefully dissected, weighed, and stored at -80ºC until use. 16 rats were used in this study. On day 21, 8 rats per group were acutely stimulated for 30 min with leptin (n = 4) or PBS infused (n = 4).

3.2 How were these groups determined?

R3.2. Animal groups and sample sizes were determined based both on our extensive prior experience with this experimental model and on the information obtained from the sample size calculation performed before the initiation of the study (see further details below in Methods, comment 9: Statistical Analysis).

3.3 How was long was the fast?

R3.3. As stated above, rats were fasted overnight (16h).

4. Line 159 - How was this dose determined? If this was informed by a previous study, please provide a reference

R4. The acute icv leptin dose (20 ng) was selected to induce a robust yet submaximal STAT3 phosphorylation response while preserving dynamic range for detecting altered leptin responsiveness. In this sense, previous studies reported that acute icv leptin (40 ng) injection increased hypothalamic STAT3 phosphorylation (pY705-STAT3) by ~ 4.5 fold in six-month-old male F344 x Brow Norway control rats, but only ~ 3.3-fold in rats overexpressing for 87 days a leptin receptor antagonist in the hypothalamus via recombinant adeno-associated virus (rAAV) gene delivered (Matheny et al., 2009), representing a ~ 27% reduction compared to control rats. Based on this data and knowing that acute brain leptin (100 ng) injection induced near- maximal hypothalamic STAT3 phosphorylation (Scarpace et al., 2001), we performed a preliminary study analyzing the effect of two doses of leptin under our experimental setting, submaximal

(20 ng) and near-maximal (100 ng). The results showed that in SLA-infused rats hypothalamic STAT3 phosphorylation was reduced ~ 40% in response to acute submaximal leptin injection (20 ng), whereas there was not a significant difference in STAT3 phosphorylation in PBS- and SLA-infused rats in response to an acute brain leptin (100 ng) injection.

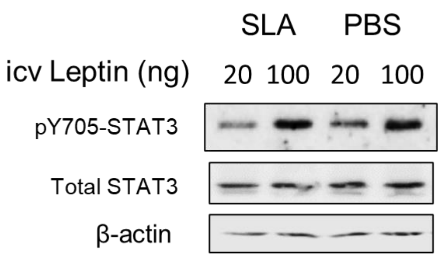

These results suggest that near-saturating receptor occupancy can produce ceiling effects, limiting the ability to detect competitive antagonism or partial reductions in leptin signaling. Hence, by using a lower leptin dose (20 ng) within the dynamic range of the dose–response curve, we increased sensitivity to detect differences in STAT3 activation between PBS- and SLA-infused cohorts in response to leptin stimulation.

5. Line 163 - again, justification of dose required

R5. The glucagon dose was selected to achieve maximal phosphorylation of the transcription factor CREB (cAMP response element-binding protein), as reported by us in previous studies (Bozadjieva et al., 2021; Merino et al., 2022). This information has been added in the Methods section as follows: The glucagon dose was selected to achieve maximal CREB phosphorylation as reported previously (Bozadjieva *et al*., 2021; Merino *et al*., 2022).

6. Line 164 - a brief description of the method (In addition to reference to previous study) would be helpful

R6. As suggested, we have included the following information in Methods section as follows: ….. the liver glucagon signaling activation by measuring canonical phosphorylation of the transcription factor CREB (cAMP response element-binding protein).

7. Line 166 - Were the animals anesthetized or euthanised by CO2 injection (with death confirmed by decapitation)?

R7. We appreciate the Referee's comment. We have now clarified this point in the revised version of the manuscript as follows: After ending the treatment, rats in the fasted state were placed in a euthanasia chamber and euthanised by CO_2 inhalation, with death confirmed by decapitation.

8. Line 219 (Gene expression analysis) - can the authors confirm that the selection of housekeeper genes followed the approved methodology? For the SYBR-green analyses - note that only 1 housekeeper was used, which is contrary to recommendations, so additional justification for this should be provided.

R8. We completely agree with the referee´s concern about the approved methodology regarding the number of housekeeping genes for the SYBR-Green analysis. Although it is recommended to use at least two or three housekeeping genes to ensure proper nor-

malization, many studies in the literature still validate gene expression analysis using only a single housekeeping gene. Besides, to rule out the possibility that the expression levels of *18S rRNA*, which we use as a single housekeeper gene, may vary depending on our experimental conditions (rats infused intracerebroventricularly with PBS or SLA), we routinely compared Ct values of *18S rRNA* in the hypothalamus and liver across different experimental cohorts of PBS *vs* SLA infused rats. The table shows *18S rRNA* amplification data used for the determination of Ct values in experimental gene analysis, performed with varying cDNA input amounts (10 ng or 50 ng).

PBS lipid metab	SLA lipid metab	PBS ObRb	SLA ObRb	PBS Circadian	SLA Circadian	PBS Acox	SLA Acox
Ct	Ct	Ct	Ct	Ct	Ct	Ct	Ct
18S rRNA	18S rRNA	18S rRNA	18S rRNA	18S rRNA	18S rRNA	18S rRNA	18S rRNA
18,86	17,45	15,99	15,65	15,30	15,06	19,25	18,35
19,39	17,40	16,02	15,60	15,32	14,98	20,29	17,71
17,56	17,08	15,96	15,10	15,10	14,61	18,28	20,02
17,31	16,76	15,70	15,16	14,94	14,51	19,21	17,71
17,65	17,10	15,50	15,57	14,82	14,94	17,38	18,06
17,14	17,24	15,15	15,55	14,78	14,94	18,13	18,18
16,88	16,93	14,94	14,80	14,37	14,36	16,92	16,94
16,83	17,16	15,02	14,83	14,40	14,35	17,61	17,17
17,34	16,89	15,15	14,85	14,53	14,29	17,01	16,63
17,23	17,29	15,20	14,89	14,59	14,35	17,84	17,01
17,55	17,48	14,97	14,91	14,43	14,44	16,66	16,44
17,52	17,49	14,98	15,04	14,46	14,54	16,79	16,38
Mean	Mean	Mean	Mean	Mean	Mean	Mean	Mean
17,61	17,19	15,39	15,17	14,76	14,62	17,95	17,55
Std Desv	Std Desv	Std Desv	Std Desv	Std Desv	Std Desv	Std Desv	Std Desv
0,76	0,24	0,42	0,33	0,34	0,28	1,14	1,03

p = 0.100 (1) p = 0.143 (1) p = 0.265 (1) p = 0.308 (2)

(1) Mann-Whitney test (2) Unpaired two-tailed Student´s t test

In addition, primers used for SYBR-Green One-Step real-time PCR were validated for specificity using melting curve analysis. This information has now been included in the revised manuscript as follows: Primers were validated for specificity using melting curve analysis. Relative gene expression levels were quantified using the ΔΔCT method, with fold changes calculated between experimental and control groups. Expression values were normalized to the mean expression of the housekeeping gene *18S rRNA*, which remained stable across all groups.

As an example, melting validation curves of a few primers used for gene expression analysis are shown for different samples in duplicate from n = 6 PBS-infused and n = 6 SLA-infused rats

18S rRNA

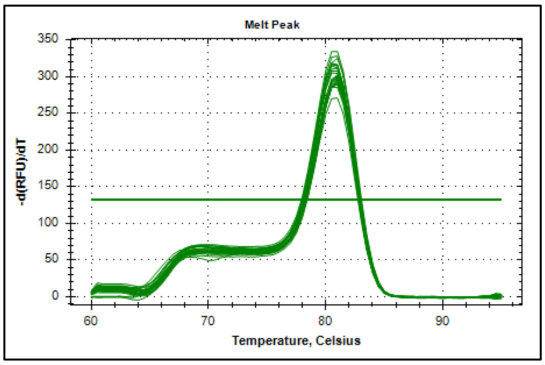

Crct2

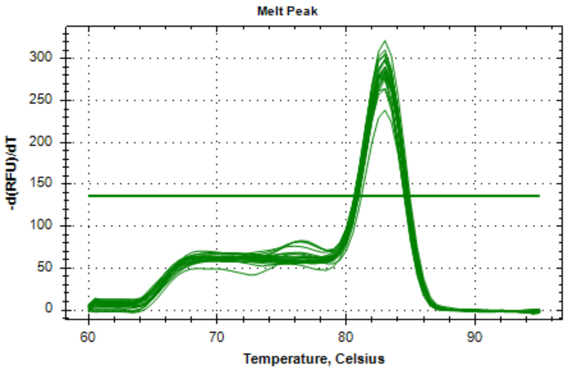

Lat 3

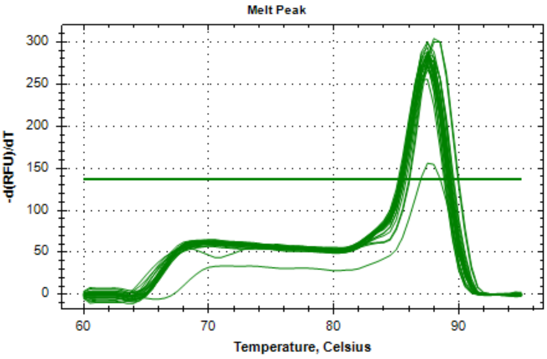

E1alpha

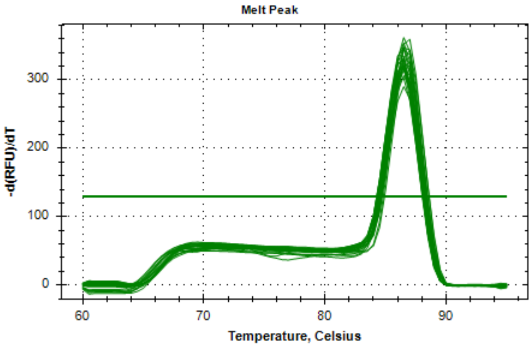

Ppm1k

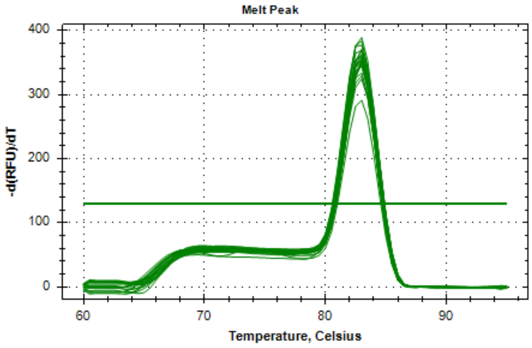

E1beta

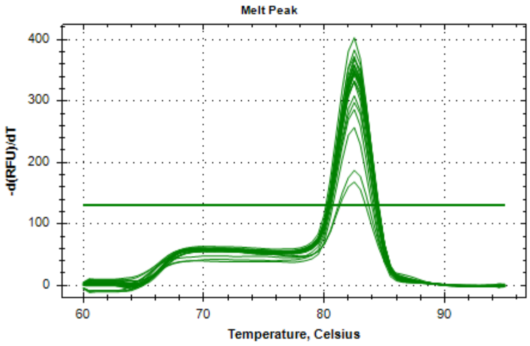

9. Statistical Analyses - why were no a priori power calculations carried out (this is typically a requirement for animal ethics applications/approvals)? Have the authors confirmed post-hoc that they had sufficient statistical power to support their conclusions?

R9. As mentioned earlier in response to your general comments, as required for animal ethic-application/approval, *a priori* sample size calculation was performed based on prior data from our group in this experimental model, a minimum difference of 125 pmol/L in circulating insulin levels between groups was considered biologically relevant, assuming a standard deviation of 50 pmol/L. Additionally, a 10% attrition rate was considered to account for potential losses and variability across the different outcome measures evaluated in the project. Under these assumptions, 6 animals per group were required.

The final number of animals included per group met the predefined sample size established in the a priori calculation. A post-hoc power analysis was not initially performed. However, in response to the reviewer's request, we have now performed post-hoc power analyses using G*Power. For serum insulin, based on the observed values (PBS: 126.7 pmol/L, SD 24.9; SLA: 161.32 pmol/L, SD 23.2), the calculated post-hoc power was 0.64. For blood glucose levels (mg/dL), using the observed values (PBS: 100.17 mg/dL, SD 5.52; SLA: 125 mg/dL, SD 14.92), the calculated post-hoc power was 0.98

While a conventional threshold of 0.8 is often cited as desirable (Cohen, 1992), values below this (such as ~0.64) do not necessarily invalidate the observed differences.

It is important to note that the interpretation of post-hoc (observed) power has been extensively discussed in the statistical literature. Several methodological publications have noted limitations of post-hoc power analysis. Specifically, that observed power estimates are dependent on the observed data and do not provide independent information about the true power of a study, and therefore should be interpreted with caution (e.g., Heinsberg & Weeks, 2022; Zhang et al., 2019; Heckman, 2022; methodology reviews on retrospective power)

For this reason, emphasis has been recommended on reporting effect sizes and confidence intervals rather than relying on post-hoc power as a measure of result validity (Nakagawa & Cuthill, 2007). Therefore, while the glucose comparison demonstrates high statistical power (0.98), the insulin comparison (0.64) should be interpreted considering its observed effect size, associated variability, and confidence intervals.

RESULTS

10. The results section includes quite a bit of explanation about rationale for certain experiments and/or interpretation of the result, some of which is somewhat repetitive - suggest reducing/deleting (e.g. Line 244 - 247, Line 284 - 285, Line 320 - 322)

R10. As suggested by the Referee, the sentences have been shortened.

11. Sufficient to show significance to 3 sig figures

R11. Exact *P* values have been included in all figures and tables.

12. Line 346 - typo 'an increased Bdk/Ppm1k ratio' should be 'and increased....'?

R12. We would like to thank you for finding the typo which has been amended.

13. Figures need to be labelled at the top of the page

R13. Done.

14. There are a lot of Figures/graphs - would consider showing some of this information in a table form instead, and reserve graphs/figures for the key findings (for added emphasis)

R14. Given the integrative physiological nature of the study and the number of independent metabolic systems examined, we consider the current Figures structure appropriate. Nevertheless, as suggested by the Referee, selected data from the Figures are now presented in Table 1. Effects of icv SLA infusion on the biological characteristics of the rats.

DISCUSSION

15. The discussion is generally well-written, however the organisation of the paragraphs makes is confusing at times. Suggest reviewing this, and ensuring that the interpretation of the relevant findings and the implications/relevance of these are included in the same paragraph.

R15. The Discussion has been reorganized so that experimental findings, their interpretation, and their physiological relevance are presented within the same paragraphs, improving clarity and logical flow.

In addition, we now include a hierarchical conceptual model (Figure 10) to visually integrate central, pancreatic, and hepatic findings, thereby improving coherence between experimental observations and their physiological interpretation.

16. Limitations of the study should also include reference to the animal numbers and statistical power (note earlier comments). Some of the gene expression graphs seem to suggest that there are only 3-4 animals per group (e.g. PPARalpha and Hgl mRNA in Figure 6. Is this really sufficient to draw robust conclusions?

R16. Thank you for your comment regarding the effective animal numbers and statistical power for the gene expression analyses, particularly *PPARα* and *Htgl* mRNA in Figure 6 (now Figure 7, in the revised version of the manuscript).

We acknowledge that for some qPCR endpoints, the effective sample size was n = 3–4 animals per group, due to exclusions following objective quality control/outlier procedures applied to the molecular dataset.

In response to your comment on robustness, we additionally quantified effect sizes and achieved power (G*Power, $\alpha = 0.05$, two-tailed). For *PPARα* (n = 4 per group), the observed effect size was large (Cohen's d ≈ 1.95), with achieved power of 0.63. While

this is below the conventional 0.8 threshold, the magnitude of the effect is substantial and consistent with the associated physiological and biochemical findings reported in the study.

For *Htgl* (n = 3 per group), the observed means were: PBS: 0.967 (SD = 0.067); SLA: 1.857 (SD = 0.038). The between-group difference was 0.89 units, with a pooled SD of 0.054, yielding a very large effect size (Cohen's d ≈ 16.4) and achieved power approaching 1.0. The 95% confidence interval for the difference was approximately [0.77, 1.01], indicating a marked separation between groups, despite the limited sample size. As recommended in the methodological literature, we interpret retrospective power cautiously and prioritize effect sizes.

Nevertheless, in response to the Referee´s comment, we have included new data from two additional animals per group in the revised version of the manuscript, resulting in a final sample size of six for *PPARα* and five for *Htgl* rats per group. As a result, the *post hoc* analysis achieved a statistical power of 0.79 for *PPARα* and 0.99 for *Htgl.*

Finally, we would like to appreciate the Referee's comment, which has improved the clarity and transparency of the statistical reporting in our manuscript.

References in response to Referee 1

Bozadjieva, K.N., Lubaczeuski, C., Blandino-Rosano, M., Blandino-Rosano, Barker, G., Gittes, G.K., Caicedo, A. & Bernal-Mizrachi, E. (2021). Glucagon Resistance and Decreased Susceptibility to Diabetes in a Model of Chronic Hyperglucagonemia. *Diabetes*, **70**(2), 477–491.

Cohen, J. (1992). A power primer. *Psychological Bulletin*, **112**(1), 155-159.

Heckman, M.G., Davis, J.M. 3rd & Crowson, C.S. (2022). Post Hoc Power Calculations: An Inappropriate Method for Interpreting the Findings of a Research Study. *Journal Rheumatology*, **49**(8), 867-870.

Heinsberg, L.W. & Weeks, D.E. (2022). Post hoc power is not informative. *Genetic Epidemiology*, **46**(7), 390-394.

Matheny, M., Zhang, Y., Shapiro, A., Tümer, N. & Scarpace, P.S. (2009). Central overexpression of leptin antagonist reduces wheel running and underscores importance of endogenous leptin receptor activity in energy homeostasis. *American Journal of Physiology Regulatory, Integrative and Comparative Physiology*, **297**(5), R1254-1261.

Mazuecos, L., Artigas-Jerónimo, S., Pintado, C., Gómez, O., Rubio, B., Arribas, C., Andrés, A., Villar, M. & Gallardo, N. (2024). Central leptin signaling deficiency induced by leptin receptor antagonist leads to hypothalamic proteomic remodeling. *Life Sciences*, **346**, 122649.

Merino, B., Casanueva-Álvarez, E., Quesada, I., González-Casimiro, C.M., Fernández-Díaz, C.M., Postigo-Casado, T., Leissring, M.A., Kaestner, K.H., Perdomo, G. & Cózar-Castellano, I. (2022). Insulin-degrading enzyme ablation in mouse pancreatic alpha cells triggers cell proliferation, hyperplasia and glucagon secretion dysregulation. *Diabetologia*, **65**(8), 1375-1389.

Nakagawa, S. & Cuthill, I.C. (2007). Effect size, confidence interval and statistical significance: a practical guide for biologists. *Biological Reviews*, **82**(4):591-605.

Scarpace, P.J., Matheny, M. & Tümer, N. (2001). Hypothalamic leptin resistance is associated with impaired leptin signal transduction in aged obese rats. *Neuroscience*, **104**(4):1111-1117.

Zhang, Y., Hedo, R., Rivera, A., Rull, R., Richardson, S. & Tu, X.M. (2019). Post how power analysis: is it an informative and meaningful analysis? *General Psychiatry*, **32**(4):e100069.

Referee #2

The paper addressed an important physiologic question if sustained attenuation of leptin receptor signaling in the brain in lean rats induces metabolic dysfunction. Adult rats received chronic IVC infusions of a rat-specific leptin receptor antagonist SLA vs vehicle for 21 day. The infusions consistently suppressed pSTAT3 signaling in the hypothalamus increasing Agrp and decreasing Socs3 and POMC gene expression, increased visceral adiposity, food intake, induced hepatic steatosis and pancreatic lipid accumulation, hyperinsulinemia and pancreatic lipid accumulation and hyperglucagonemia. Interestingly, hyperglucagonemia did not induce gluconeogenesis enzymes. In contrary, gluconeogenesis genes and CREB phosphorylation decreased, which suggest hepatic glucagon resistance. There was also evidence of disrupted glucagon-amino acid signaling manifested by elevated serum total and branched-chain amino acid levels. All these changes occurred without any changes in serum leptin and minimal increase in body weight without changes in the Obesity Lee index (Rat BMI). The authors conclude that attenuation of brain leptin signaling leads to metabolic dysfunction prior to obesity development.

The paper represents an important advance in our knowledge and it is scientifically sound.

We would like to thank the Referee for their kind comments and constructive suggestions on the work, all of which are valuable and helpful for revising and improving the manuscript.

Nevertheless, there are several important issues need to be addressed to increase these findings impact:

Major critique: 1) It would be important to assess energy expenditure and overall activity in view of the profound metabolic shifts. VO2/VCO2 and RER measurements would strengthen the manuscript. Did the core body temperature change?

R1. We agree that assessment of energy expenditure and activity would provide additional insight. These measurements were already performed in this model and have recently been submitted for abstract presentation at ENDO 2026 Congress. Nevertheless, the present manuscript was intentionally designed to address a distinct biological question, namely, how impaired central leptin signaling disrupts glucose, lipid, amino acid, and pancreatic endocrine homeostasis in lean animals, independent of

energy expenditure measurements. We believe that the conclusions of this study are fully supported by the data presented. The calorimetric and behavioral findings will be reported separately to avoid overlapping, maintain a clear mechanistic focus, and ensure conceptual clarity between the manuscripts.

However, in response to the Referee´s comments, we summarize the results recently submitted for abstract presentation: energy expenditure did not differ significantly between both groups of rats; however, energy expenditure (EE) amplitude decreased in SLA-infused compared with PBS-infused rats. SLA infusion reduced core body temperature (included in the new Table 1) and increased the respiratory exchange ratio, indicating preferential carbohydrate utilization over lipids, supporting that SLA impairs the anti-steatotic effects of central leptin. The slight body weight gain was attributed to reduced locomotor activity, altered food reward pathways, and changes in circadian rhythmicity.

Finally, we would like to note that, in accordance with the embargo policy, all abstracts presented at the ENDO 2026 Congress must not be publicly released until the scheduled date and time of presentation.

2) Did the muscle mass decrease after SLA treatment? Were there any changes in subcutaneous adiposity and the amount of brown fat?

R2. We would like to thank the Referee for this insightful comment. Although this is an interesting point, unfortunately, we were unable to analyze these parameters due to a lack of Core facilities at our Institution, which are necessary for assessing total body fat, lean body mass, and subcutaneous adiposity. In previous studies, we analyzed total body fat and lean body mass by dual energy X-ray absorptiometry (DXA) (Escrivá et al., 2007); However, this was a very challenging task due to the need to handle animals outside our animal facilities and the absence of appropriate equipment at our institution.

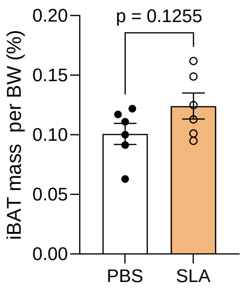

In icv SLA-infused rats there was a modest increase in interscapular brown adipose tissue mass (iBAT) (new Table 1). This was accompanied by iBAT whitening and reduced average temperature (PBS -infused rats 31.88 ± 0.15 ºC, n = 4; SLA-infused rats 31.15 ± 0.16 ºC, n = 4; p = 0.0006), indicative of impaired thermogenic function.

3) There was a slight increase in body weight without any change in the Obesity Lee Index: was there any change in body length?

R3. There was not a significant change in body length between the two groups (PBS-infused rats: 22.95 ± 0.71 cm, n = 8; SLA-infused rats, 23.50 ± 0.45 cm, n = 8; p = 0.0898). The results corresponding to the Obesity Lee Index have now been included in the revised version of the manuscript in Table 1.

4) How do the authors explain fasting hyperglycemia after SLA treatment despite suppressed gluconeogenesis?

R4. We thank the reviewer for this insightful question. The coexistence of fasting hyperglycemia with reduced gluconeogenic gene expression and attenuated hepatic CREB phosphorylation indicates that elevated fasting glucose is not driven by increased hepatic gluconeogenesis per se. Rather, these findings point to impaired hierarchical integration of peripheral metabolic signals.

Peripheral signals arising from the gut and hepatoportal region can regulate systemic glucose homeostasis through neural and hormonal pathways without requiring transcriptional activation of gluconeogenesis (Joly-Amado et al., 2022). Accordingly, chronic disruption of central signaling in our model likely impairs central interpretation of peripheral metabolic inputs, leading to inappropriate regulation of hepatic glucose output, glycogen handling, and peripheral glucose disposal. This framework provides a unifying explanation for the dissociation between circulating glucose levels and hepatic gluconeogenic gene expression observed in our study.

Consistent with these observations, we propose a hierarchical model (Figure 10) in the revised version of the manuscript in which impaired central energy-sensing integration acts as the primary driver of metabolic dysregulation, with pancreatic endocrine remodeling and hepatic dysfunction emerging as parallel downstream effectors rather than sequential causal events. This schematic model provides a visual framework to explain the dissociation between fasting hyperglycemia and reduced hepatic gluconeogenic gene expression observed in SLA-treated rats.

5) Statistical Analysis - did you check your data for normality? If the data are not normally distributed, non-parametric statistics should be used

R5. We thank the Referee for this observation which give us the opportunity to provide further details on the statistical analysis. Normal distribution of the data was checked with the Shapiro-Wilk test. When the distribution of data was normal, differences between predefined experimental groups were determined unpaired two-tailed Student's *t*-test followed by Welch´s correction, as appropriate or two-way ANOVA followed by Holm–Šidák multiple-comparison testing. Non-parametric data were analysed by using the Mann-Whitney test. Besides, to accomplish the goals of the statistical policy, all data are now expressed as means ± SD. This information has been included in the revised version of the manuscript.

Minor critique:

1) Can you describe how you estimated liver glucagon signaling rather than providing a reference? - Line 164

R1. As suggested by the Referee, we have added this information in the revised version of the manuscript as follows: we analyze the liver glucagon signaling activation by measuring canonical phosphorylation of the transcription factor CREB (cAMP response element-binding protein).

2) In the results - line 250, include a reference to Fig. 1A

R2. We would like to thank the Referee for pointing out this error, which has now been amended. In the revised version of the manuscript appears as Fig. 2A.

3) Line 264-265 - change to 'no significant change in glycogen and corticosterone levels'

R3. As suggested, we have modified the sentence as follows: In addition, SLA-infused rats displayed no significant changes in hepatic glycogen content and circulating corticosterone levels ….

4) Line 280: the C-peptide/insulin ratio reflects hepatic insulin clearance not IR. Please re-word

R4. We did not reword this because it was already stated in the previous version of the manuscript.

5) Line 325 - Add that TNF-a expression is decreased. It suggests that the data on a pro-inflammatory effect are equivocal

R5. As suggested by the Referee, we have included this information in the Results section as follows: Although the expression of *Tnfα* was reduced by SLA infusion, markers of hepatic inflammation and stress, including Il1β and Ccl5, were elevated (Fig. 7C). In parallel, Got1 expression was increased (Fig. 7D), consistent with early stress-associated and profibrotic signaling rather than a classical TNFα-driven inflammatory response (37–39).

References in response to Referee 2

Escrivá, F., Gavete, M.L., Fermín, Y., Pérez, C., Gallardo, N., Alvarez, C., Andrés, A., Ros, M. & Carrascosa, J.M. (2007). Effect of age and moderate food restriction on insulin sensitivity in Wistar rats: role of adiposity. *Journal of Endocrinology*, **194**(1), 131-141.

Joly-Amado, A., Soty, M., Philippe, E., Lacombe, A., Castel, J., Pillot, B., Vily-Petit, J., Zitoun, C., Mithieux, G. & Magnan, C. (2022). Portal Glucose Infusion, Afferent Nerve Fibers, and Glucose and Insulin Tolerance of Insulin-Resistant Rats. Journal of Nutrition, **152**(8), 1862-1871.

Dear Dr ANDRES,

Re: JP-RP-2026-290832R1 "**Chronic Attenuation of Brain Leptin Signaling Is Associated with Early Metabolic Dysfunction in Lean Rats**" by CRISTINA PINTADO, LORENA MAZUECOS, ÓSCAR GÓMEZ-TORRES, BEATRIZ MERINO, ELENA CASANUEVA-ÁLVAREZ, BLANCA RUBIO, EMMA BURGOS-RAMOS, SARA ARTIGAS-JERÓNIMO, EDUARDO MOLTÓ, INÉS MARÍA RAMOS, JUSTA MARÍA POVEDA, GERMÁN PERDOMO, IRENE CÓZAR-CASTELLANO, CARMEN ARRIBAS, ERNESTO BERNAL-MIZRACHI, ANTONIO ANDRES, and NILDA GALLARDO

Thank you for submitting your manuscript to The Journal of Physiology. It has been assessed by a Reviewing Editor and by 2 expert referees and we are pleased to tell you that it is acceptable for publication following satisfactory revision.

The referee reports are copied at the end of this email.

Please address all the points raised and incorporate all requested revisions or explain in your Response to Referees why a change has not been made. We hope you will find the comments helpful and that you will be able to return your revised manuscript within 4 weeks. If you require longer than this, please contact journal staff: jp@physoc.org.

Your revised manuscript should be submitted online using the link in your Author Tasks Link Not Available. This link is accessible via your account as Corresponding Author; it is not available to your co-authors. If this presents a problem, please contact journal staff (jp@physoc.org). Image files from the previous version are retained on the system. Please ensure you replace or remove any files that are being revised.

If you do not wish to submit a revised version of your manuscript, you must inform our journal staff (jp@physoc.org) or reply to this email to request withdrawal. Please note that a manuscript must be formally withdrawn from the peer review process at one journal before it may be submitted to another journal.

TRANSPARENT PEER REVIEW POLICY: To improve the transparency of its peer review process The Journal of Physiology publishes online, as supporting information, the peer review history of all articles accepted for publication. Readers will have access to decision letters, including Editors' comments and referee reports, for each version of the manuscript, as well as any author responses to peer review comments. Referees can decide whether or not they wish to be named on the peer review history document.

ABSTRACT FIGURES: Authors are expected to use The Journal's premium BioRender account to create/redraw their Abstract Figures. Information on how to access this account is here: https://physoc.onlinelibrary.wiley.com/journal/14697793/biorender-access.

This will enable Authors to create and download high-resolution figures. If authors have used the free BioRender service, they can use the instructions provided in the link above to download a high-resolution version suitable for publication.

The link provided should only be used for the purposes of this submission. Authors will be charged for figures created on this account if they are not related to this manuscript submission.

LANGUAGE EDITING AND SUPPORT FOR PUBLICATION: If you would like help with English language editing, or other article preparation support, Wiley Editing Services offers expert help, including English Language Editing, as well as translation, manuscript formatting, and figure formatting at www.wileyauthors.com/eeo/preparation. You can also find resources for Preparing Your Article for general guidance about writing and preparing your manuscript at www.wileyauthors.com/eeo/prepresources.

RIGOUR AND REPRODUCIBILITY: We recommend authors consult The Journal's Rigour and Reproducibility requirements to ensure that their manuscript is suitable for publication: https://physoc.onlinelibrary.wiley.com/pb-assets/hub-assets/physoc/documents/TJP-Rigour-and-Reproducibility-Requirements.pdf

REVISION CHECKLIST:

Check that your Methods section conforms to journal policy: https://jp.msubmit.net/cgi-bin/main.plex?form_type=display_requirements#methods

Check that data presented conforms to the statistics policy: https://jp.msubmit.net/cgi-bin/main.plex?form_type=display_requirements#statistics

Upload a full Response to Referees file. To create your 'Response to Referees' copy all the reports, including any comments from the Senior and Reviewing Editors, into a Microsoft Word, or similar, file and respond to each point, using font or background colour to distinguish comments and responses and upload as the required file type.

Please upload two versions of your manuscript text: one with all relevant changes highlighted and one clean version with no changes tracked. The manuscript file should include all tables and figure legends, but each figure/graph should be uploaded

as separate, high-resolution files. The journal is now integrated with Wiley's Image Checking service. For further details, see: https://www.wiley.com/en-us/network/publishing/research-publishing/trending-stories/upholding-image-integrity-wileys-image-screening-service

You may also upload:

- 'Potential Cover Art' for consideration as the issue's cover image
- Appropriate Supporting Information (Video, audio or data set: see https://jp.msubmit.net/cgi-bin/main.plex?form_type=display_requirements#supp).

We look forward to receiving your revised submission.

If you have any queries, please reply to this email and we will be pleased to advise.

Yours sincerely,

Bettina Mittendorfer
Senior Editor
The Journal of Physiology

REQUIRED ITEMS

EDITOR COMMENTS

Both reviewers were pleased with the revisions and felt that the authors have adequately addressed most of the comments. One of the reviewers provided additional feedback regarding statistical power which the authors should address before final acceptance of the paper. The number of figures as large but appropriate and does not have to be altered.

REFEREE COMMENTS

Referee #1:

I am generally comfortable with the authors responses to my previous comments. I appreciate the authors work to include additional animals in some of the analyses but do remain somewhat concerned by the low animal numbers per group in a number of the analyses (especially gene expression), and the limitations this places on being able to draw robust conclusions. As a result, I suggest that some additional text be added to the limitations section of the discussion to acknowledge this. I also still feel that the number of figures is excessive (and a bit overwhelming!), so suggest potentially moving more of this information into Table form and/or including less significant data to an appendix.

Otherwise, I have no further comments.

Referee #2:

This is an impactful paper. The experimental design is rigorous. My critique has been addressed.

END OF COMMENTS

RESPONSE TO REFEREES

EDITOR COMMENTS

Both reviewers were pleased with the revisions and felt that the authors have adequately addressed most of the comments. One of the reviewers provided additional feedback regarding statistical power which the authors should address before final acceptance of the paper. The number of figures is large but appropriate and does not have to be altered.

We thank the Editor and the Referees for their kind comments and careful evaluation of our manuscript, which have helped us improve the clarity and transparency of our study. Consequently, we have included a new sentence in the Discussion to explicitly acknowledge the limitations related to sample size, particularly in some gene expression analyses, as suggested by Referee #1. All changes included in the revised version of the manuscript are highlighted in red font.

REFEREE COMMENTS

Referee #1

I am generally comfortable with the authors responses to my previous comments. I appreciate the authors work to include additional animals in some of the analyses but do remain somewhat concerned by the low animal numbers per group in a number of the analyses (especially gene expression), and the limitations this places on being able to draw robust conclusions. As a result, I suggest that some additional text be added to the limitations section of the discussion to acknowledge this.

As suggested, we have included a new sentence in the Discussion section to explicitly acknowledge the limitations related to sample size, particularly in certain gene expression analyses. The new sentence in the revised version of the manuscript is as follows:

"An additional limitation relates to the sample size in some gene expression analyses, where a low number of animals per group may reduce statistical power. Although these analyses were conducted in independent cohorts and yielded consistent directional changes across multiple endpoints, the relatively small sample size in these assays should be considered when interpreting the findings. Future studies with larger sample sizes will be important to confirm these observations."

I also still feel that the number of figures is excessive (and a bit overwhelming!), so suggest potentially moving more of this information into Table form and/or including less significant data to an appendix.

Thank you for your suggestion. Nevertheless, given the integrative physiological nature of the study and the need to present multiple independent metabolic endpoints, we believe that the current figure structure facilitates interpretation and effectively supports the overall conclusions. In the previous manuscript version, some results were presented in a Table, while less critical data were relegated to an Appendix.

Otherwise, I have no further comments.

We thank the Referee for their kind comments and constructive suggestions on this work.

Referee #2

This is an impactful paper. The experimental design is rigorous. My critique has been addressed.

Thank you very much for your kind comments; they are truly appreciated.

Dear Professor ANDRES,

Re: JP-RP-2026-290832R2 "**Chronic Attenuation of Brain Leptin Signaling Is Associated with Early Metabolic Dysfunction in Lean Rats**" by CRISTINA PINTADO, LORENA MAZUECOS, ÓSCAR GÓMEZ-TORRES, BEATRIZ MERINO, ELENA CASANUEVA-ÁLVAREZ, BLANCA RUBIO, EMMA BURGOS-RAMOS, SARA ARTIGAS-JERÓNIMO, EDUARDO MOLTÓ, INÉS MARÍA RAMOS, JUSTA MARÍA POVEDA, GERMÁN PERDOMO, IRENE CÓZAR-CASTELLANO, CARMEN ARRIBAS, ERNESTO BERNAL-MIZRACHI, ANTONIO ANDRES, and NILDA GALLARDO

We are pleased to tell you that your paper has been accepted for publication in The Journal of Physiology.

The last Word (or similar) version of the manuscript provided will be used by the Production Editor to prepare your proof. When this is ready you will receive an email containing a link to Wiley's Online Proofing System. The proof should be thoroughly checked and corrected as promptly as possible.

Authors should note that it is too late at this point to offer corrections prior to proofreading. Major corrections at proof stage, such as changes to figures, will be referred to the Editors for approval before they can be incorporated. Only minor changes, such as to style and consistency, should be made at proof stage. Changes that need to be made after proof stage will usually require a formal correction notice.

All queries at proof stage should be sent to: TJP@wiley.com.

Following copyediting and typesetting the accepted version will be published online.

Yours sincerely,

Bettina Mittendorfer
Senior Editor
The Journal of Physiology

IMPORTANT POINTS TO NOTE FOLLOWING ACCEPTANCE OF YOUR PAPER:

• IMPORTANT NOTICE ABOUT OPEN ACCESS: To assist authors whose funding agencies mandate immediate public access to published research findings, The Journal of Physiology allows authors to pay an Open Access (OA) fee to have their papers made freely available immediately on publication.

The Corresponding Author will receive an email from Wiley with details on how to register or log in to Wiley Authors where you will be able to place an order.

• You can check if your funder or institution has a Wiley Open Access Account here:
https://authors.wiley.com/author-resources/Journal-Authors/open-access/author-compliance-tool.html

• TRANSPARENT PEER REVIEW POLICY: To improve the transparency of its peer review process, The Journal of Physiology publishes online as supporting information the peer review history of all articles accepted for publication. Readers will have access to decision letters, including Editors' comments and referee reports, for each version of the manuscript as well as any author responses to peer review comments. Referees can decide whether or not they wish to be named on the peer review history document.

• You can help your research get the attention it deserves! Check out Wiley's free Promotion Guide for best-practice recommendations for promoting your work at: www.wileyauthors.com/eeo/guide. You can learn more about Wiley Editing Services which offers professional video, design, and writing services to create shareable video abstracts, infographics, conference posters, lay summaries, and research news stories for your research at: www.wileyauthors.com/eeo/promotion.

• If you would like to receive our 'Research Roundup', a monthly newsletter highlighting the cutting-edge research published in The Physiological Society's family of journals (The Journal of Physiology, Experimental Physiology, Physiological Reports, The Journal of Nutritional Physiology and The Journal of Precision Medicine: Health and Disease), please click this link, fill in your name and email address and select 'Research Roundup':
https://www.physoc.org/journals-and-media/membernews

REFEREE COMMENTS

Referee #1:

Thank you for the response to my previous comments, and for the additional information now included in the discussion section of the manuscript in relation to the limitation of the small number of animals included in several of the gene expression analyses. I have no further comments.
